# GapClust is a light-weight approach distinguishing rare cells from voluminous single cell expression profiles

**DOI:** 10.1038/s41467-021-24489-8

**Published:** 2021-07-07

**Authors:** Botao Fa, Ting Wei, Yuan Zhou, Luke Johnston, Xin Yuan, Yanran Ma, Yue Zhang, Zhangsheng Yu

**Affiliations:** 1 Department of Bioinformatics and Biostatistics, School of Life Sciences and Biotechnology, Shanghai Jiao Tong University, Shanghai, China; 2 SJTU-Yale Joint Centre for Biostatistics, Shanghai Jiao Tong University, Shanghai, China; 3 School of Mathematical Sciences, Shanghai Jiao Tong University, Shanghai, China; 4 Clinical Research Institute, Shanghai Jiao Tong University School of Medicine, Shanghai, China

**Keywords:** RNA sequencing, Software, Statistical methods

## Abstract

Single cell RNA sequencing (scRNA-seq) is a powerful tool in detailing the cellular landscape within complex tissues. Large-scale single cell transcriptomics provide both opportunities and challenges for identifying rare cells playing crucial roles in development and disease. Here, we develop GapClust, a light-weight algorithm to detect rare cell types from ultra-large scRNA-seq datasets with state-of-the-art speed and memory efficiency. Benchmarking on diverse experimental datasets demonstrates the superior performance of GapClust compared to other recently proposed methods. When applying our algorithm to an intestine and 68 k PBMC datasets, GapClust identifies the tuft cells and a previously unrecognised subtype of monocyte, respectively.

## Abstract (web summary)

While rare cell type identification is indispensable in single cell studies, powerful tools with high detection accuracy and computational efficiency are still lacking. Here, the authors propose a light-weight algorithm which can distinguish rare cell types from voluminous single cell expression profiles.

## Introduction

The introduction of single-cell RNA sequencing (scRNA-seq) technology has significantly boosted biomedical research in single cell resolution, which is a necessity for dissecting cellular heterogeneity within complex tissues in health and disease. With evolving throughput and efficiency, scRNA-seq datasets consisting of transcription profiles with over a million cells have been contributed^1–4^. Major cell types among these can be characterised comprehensively with toolkits such as Seurat^5^ and Scater^6^, whereas the rare cell types, making up the majority of the new cell types, remain to be uncovered by specialised approaches. Moreover, evidence suggests that rare cell populations, including circulating tumour cells^7^, endothelial progenitor cells^8,9^ and antigen specific T cells^10–12^, are instrumental in cancer pathogenesis, angiogenesis and immune response mediation in cancer and other diseases. With increasingly vast numbers of cells profiled by advanced technologies, more rare cell types can be sampled, posing opportunities and challenges for new cell type identification.

With extensive investigations, several algorithms for rare cell type detection are readily available, and eminent among these are rare cell-type identification (RaceID)^13^, GiniClust^14^, cell subtype identification from up-regulated gene sets (CellSIUS)^15^ and finder of rare entities (FiRE)^16^. RaceID appropriates information from iterative steps of unsupervised clustering with the expense of large computational costs determining abundant cell types, and therefore discovering outlier cells. GiniClust uses a more straight-forward strategy, identifying rare cell types by applying density-based clustering with informative genes, which are exclusively expressed within rare cell populations and consequently show high Gini index values. CellSIUS takes advantage of established major cell types to screen marker genes whose expression exhibits a bimodal distribution within each cluster and then aggregate these genes for one-dimension clustering to identify cluster-specific sub-populations. FiRE employs the highly efficient Sketching technique^17^ to assign each cell a hash code for multiple times and uses the populousness of a hash code as an indicator of the rareness of its resident cells. In contrast with the other three methods for discrete stratification, FiRE assigns a continuous score to each cell, and those with scores higher than the threshold are identified as rare cells. In addition, EGDE^18^, a novel approach for dimensionality reduction and feature gene extraction of scRNA-seq data, can detect rare cell groups as a side product.

Notably, the algorithms above can be used depending on the characteristics of the dataset. In growing cases, scRNA-seq datasets containing thousands of cells preclude the use of RaceID due to unrealistic amount of running time^14,16^, and GiniClust, which was reported to fail in processing expression data of over 45,000 cells^16^. Moreover, droplet-based protocols have enabled the parallel profiling of tens of thousands of single cells^1^, which are widely used due to significantly reduced per-cell cost. CellSIUS can only be applied to datasets with major cell types determined^15^, and misclassified cluster information would considerably compromise the efficacy. FiRE is more powerful in handling large scRNA-seq datasets but does not discriminate between an outlier and cells representing minor cell types, thus relies on downstream analysis to flag outlier cells and identify minor cell clusters^16^. Intuitively, EDGE can provide two-dimensional embeddings of scRNA-seq datasets to show the rare cell types^18^. However, the exact indexes of the rare cell types need to be analytically determined.

To overcome these limitations, we have developed a light-weight tool, GapClust, which achieves a balance between accuracy and efficiency when searching for the needle (rare cells) in the haystack. Instead of clustering by iterative modeling or marker genes that exhibit skewed or bimodal distributions, the design of GapClust is inspired by the observation that for a particular data point in a minor cluster *C*_*n*_ of size *n*, then the distance to its nearest neighbours outside *C*_*n*_ is far greater than to its neighbours inside *C*_*n*_ due to the gap between *C*_*n*_ and its neighbouring cluster. A second-order derivative type distance score capturing the variation of distance changes is proposed to fully utilise this gap information. With diverse simulation experiments based on multiple scRNA-seq datasets, we have demonstrated that GapClust outperforms existing methods in both precision and sensitivity in terms of rare cell detection. More importantly, superior speed and memory efficiency enable GapClust to handle vast scRNA-seq datasets with ease and manage the evolving throughput of scRNA-seq era^19^. Applications to the intestine and 68 k PBMC datasets display the capability of GapClust in rare cell type identification.

## Results

### Overview of the GapClust

**Fig. 1:**
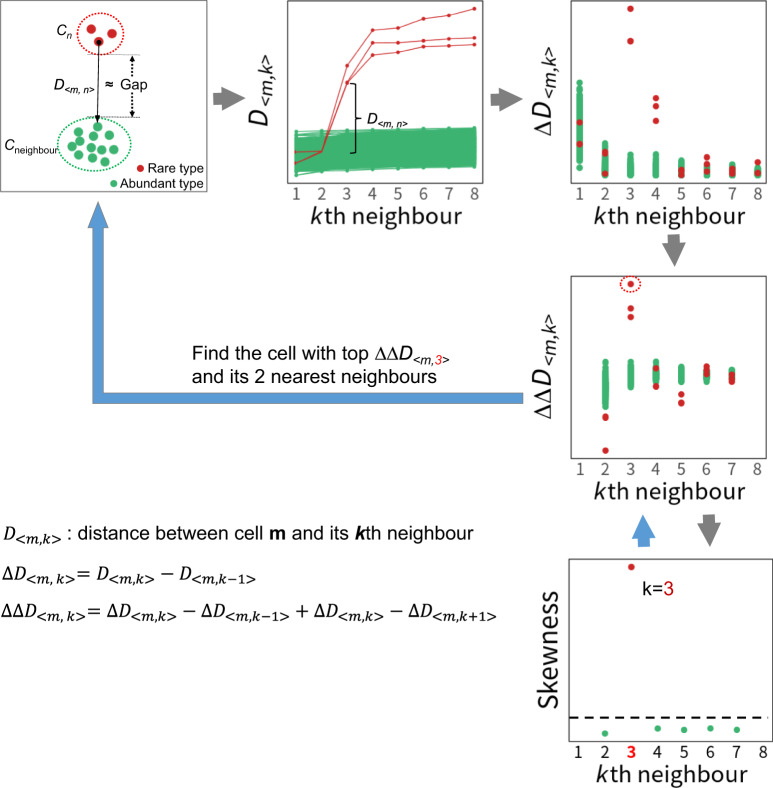
Overview of GapClust. The first step is obtaining *K* nearest neighbours for all cells. For each cell *m*, ∆*D*_*<m, k>*_ and ∆∆*D*_*<m, k>*_ can be obtained according to the formula. Then the skewness of adjusted ∆∆*D*_*<m, k>*_ values is calculated. Candidate *k* can be identified if skewness > 2. In the last step, for each candidate *k*, the cell with largest ∆∆*D*_*<m, k>*_ value among *N* cells and its *k*-1 nearest neighbours are identified and subject to filtering steps to determine the final rare cell types.

To facilitate the understanding, we use a two-dimensional representation of each cell for illustration (the first box in Fig. 1), where the clusters in different colours denote different cell types. Herein, the gap denotes the large Euclidean distance between the rare cell cluster *C*_*n*_ (red) and the neighbouring cluster *C*_neighbour_ (green) based on their expression profiles (the first box in Fig. 1). The gap is closely associated with the identification of the rare cell type *C*_*n*_, as it can be approximated by the distance between any given cell *m* in *C*_*n*_ and its nearest neighbour from *C*_neighbour_ (the first box in Fig. 1), since the gap is large relative to the minor cluster *C*_*n*_. For each cell *m* in *C*_*n*_, its first *n*-1 nearest neighbours are the remaining *n*-1 cells in *C*_*n*_, and then its *n*th nearest neighbour is from *C*_neighbour_, namely the nearest neighbour cell in *C*_neighbour_. Therefore, the gap can be estimated with the distance *D*_*<m, n>*_ between each cell *m* in *C*_*n*_ and its *n*th neighbour (the first box in Fig. 1, Supplementary Fig. 1).

As a result of the large gap relative to the minor cluster *C*_*n*_, the abrupt increase of *D*_*<m, n>*_ can be observed and used to distinguish the *n* cells of the *C*_*n*_ cell type (red) from the neighbouring abundant cell type (green) (the second box in Fig. 1). Importantly, the smaller number (*n*) of cells in *C*_*n*_, the earlier abrupt increase of *D*_*<m, n>*_ values at *n*th nearest neighbour of each cell in *C*_*n*_ can be observed. As rare cell types such as *C*_*n*_ have low abundance (small *n*), we can capture *C*_*n*_ based on the abrupt increase of *D*_*<m, n>*_ values without inquiring too many neighbours of each cell.

According to the basic mathematical theory that the first-order derivative of a variable represents the rate of change of itself with respect to another variable, this abrupt increase in *D*_*<m, n>*_ value of the cells from *C*_*n*_ can thus be captured by its considerably large first-order derivative value ∆*D*_*<m, n>*_ (the third box in Fig. 1). However, the values of ∆*D*_*<m, k>*_ where *k* is equal to 1 or close but not equal to *n* can also be relatively large, posing barriers to accurate identification of ∆*D*_*<m, n>*_ (the third box in Fig. 1).

Compared with the first-order derivative, the second-order derivative is widely used for measuring the roughness variation of a twice-differentiable curve^20^ and preferred for the identification of peaks in spectra analysis^21^. To capture the ∆*D*_*<m, n>*_ value, we design a second-order derivative like statistic ∆∆*D*_*<m, k>*_ (formula in Fig. 1), which measures the summation of the difference between the ∆*D*_*<m, k>*_ and each of the ∆*D*_*<m, k−1>*_, ∆*D*_*<m, k + 1>*_. Compared with ∆*D*_*<m, k>*_, ∆∆*D*_*<m, k>*_ can maintain the peak value of ∆∆*D*_*<m, n>*_, but shrink the values of ∆∆*D*_*<m, k>*_ when *k* is close but not equal to *n*, giving the exact location of ∆*D*_*<m, n>*_ (the fourth box in Fig. 1).

To quantitatively capture the peak location of ∆∆*D*_*<m, n>*_, we first calculate the skewness of the distribution of ∆∆*D*_*<m, k>*_ values of all cells when *k* varies from 1 to *K*, and high skewness_*<n>*_ larger than the threshold value 2 can be observed (the fifth box in Fig. 1)^22^, as large values of ∆∆*D*_*<m, n>*_ from the rare cells in *C*_*n*_ lead to right-skewed distribution. Finally, the *n* cells comprising the cell with top ∆∆*D*_*<m, n>*_ value and its *n*-1 nearest neighbours are determined as the rare cell type *C*_*n*_. The details of the algorithm are outlined in the Methods section.

### GapClust accurately detects artificially planted rare cells

To provide a proof-of-concept, we have used Splatter^23^ to simulate five scenarios of different levels of differential expression between the rare cell type and the abundant cell types. In each scenario, we generate 99 datasets comprising two major cell types of 500 cells and one rare cell type of different numbers of rare cells (2–100), with which five methods including RaceID, GiniClust, CellSIUS, FiRE and GapClust are compared in terms of detection performance. We utilised *F*_1_ score for performance evaluation concerning rare cell type identification, which reflects the balance between precision and sensitivity (Methods). The results (Supplementary Fig. 2a-e) correspond to five different settings of differential expression levels between the rare cell type and the two abundant cell types, with varying probabilities (0.1, 0.2, 0.4, 0.6 and 0.8) that a gene is differentially expressed (DE) in the rare cell group. In comparison, GapClust provides the best performance among all the methods in all simulation settings. RaceID detects the rare cells perfectly in most datasets but could still not be comparable to GapClust. Despite the high *F*_1_ scores, the performances of CellSIUS and FiRE are not stable throughout all the datasets. GiniClust displays a relatively poor capability in detecting the rare cells in most datasets. In addition, we evaluate the sensitivity and specificity of these five methods on the simulated datasets (Supplementary Fig. 3, 4), and find that GapClust can provide high sensitivity and specificity simultaneously across all datasets.

**Fig. 2:**
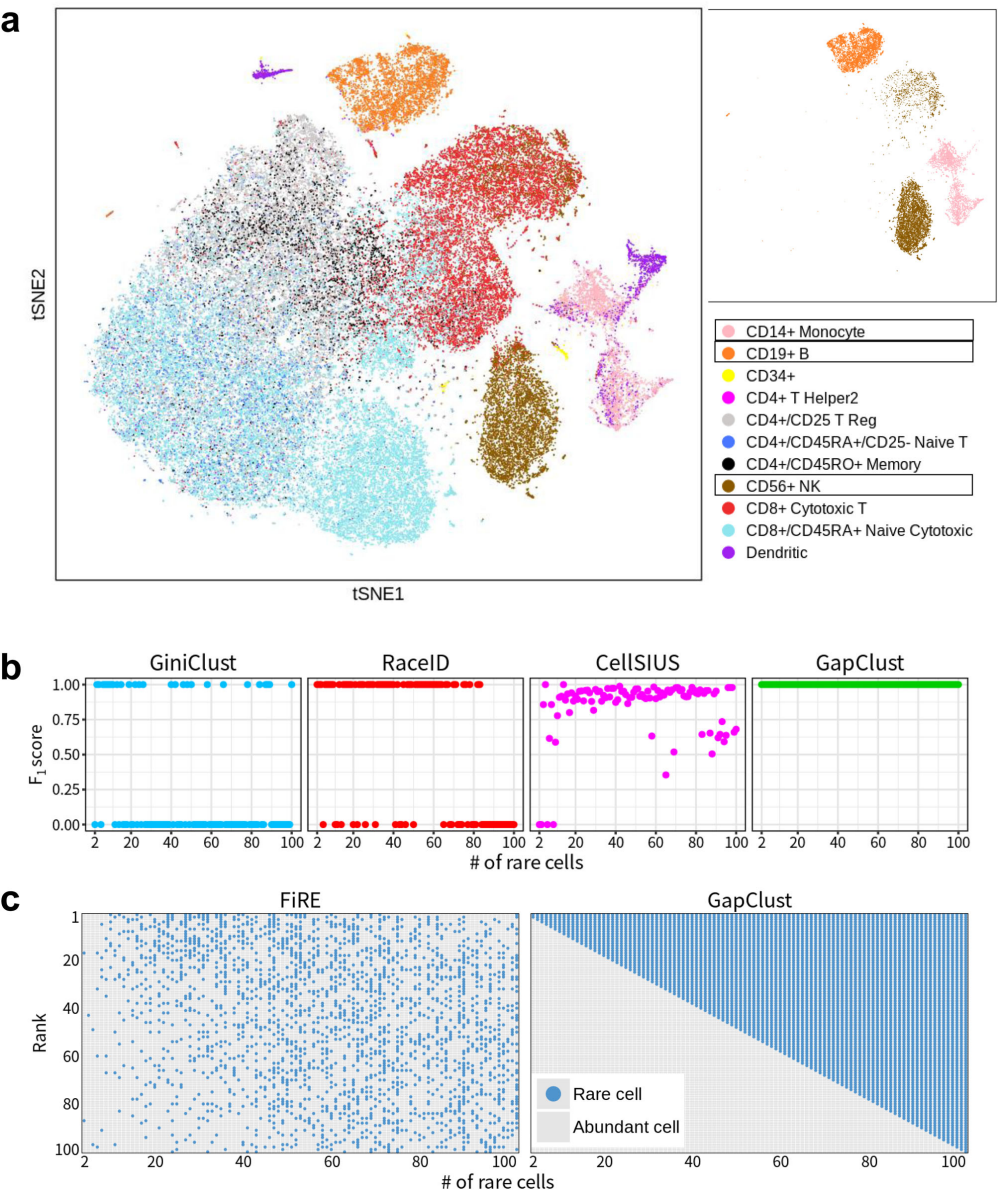
Analysis of the simulated datasets subsampled from the 68 k PBMC dataset. **a** The tSNE plot of the full dataset with reference labels (left), along with the three cell types selected for analysis (right). **b** Evaluation of the performance of different methods for rare cell type detection, quantified by *F*_1_ score. **c** Comparison of the ranking results for rare cells between GapClust and FiRE by continuous scores.

We compared the performance of GapClust to GiniClust, RaceID, CellSIUS and FiRE using a simulation-based experiment in the presence of ground truth cell-type identity. For this, we used a widely utilised public scRNA-seq dataset consisting of transcription profiles of ~68,000 peripheral blood mononuclear cells (PBMCs)^2^, which were classified into 11 sub-types. We focused on three large sub-populations: CD56 + natural killer (NK) cells, CD14 + monocytes and CD19 + B cells, whose transcriptomic profiles were distinct from one another (Fig. 2a). To mimic the rare cell phenomenon systemically, we added between 2 and 100 monocytes (0.2–10% of rare cell-type proportions) to two homogeneous populations of 500 NK cells and B cells (Methods).

Taken all cases with various numbers of rare cells together, GapClust outperformed GiniClust, RaceID, CellSIUS and FiRE in identifying the rare monocytes (Fig. 2b, and Supplementary Fig. 5). GiniClust and RaceID lost accuracy in 73 and 40% of all cases with an *F*_1_ score of zero, respectively. Despite high accuracy on average, CellSIUS could not provide perfect detection uniformly with an *F*_1_ score of 1 for most cases. FiRE performed extremely poor with the default threshold criterion of q3 + 1.5 × IQR (Supplementary Fig. 5), where q3 and IQR denote the third quartile and the interquartile range (75th percentile–25th percentile), respectively, of the number of FiRE scores across all cells. We implemented less stringent criteria of q3 + 1.0 × IQR and q3 + 0.5 × IQR, which improved the performance, yet this still was not comparable to GapClust (Supplementary Fig. 5). In addition, we evaluate the sensitivity and specificity of these five methods on the subsampled datasets (Supplementary Fig. 6) and find that GapClust can provide high sensitivity and specificity simultaneously across all datasets. Notably, GiniClust, RaceID and CellSIUS provided dichotomised predictions for rare cells, whereas GapClust and FiRE offered continuous scores and binary prediction. To eliminate the effect of the thresholding technique, we directly compared the rankings of rare cells using scores estimated by GapClust and FiRE, respectively. Consistently, rankings of monocytes by GapClust were more enriched at the extreme top of the entire cells compared with FiRE (Fig. 2c).

To further evaluate the performance of these algorithms, we reproduced another experiment by Jindal et al.^16^ using a scRNA-seq data comprising of 293T and Jurkat cells mixed in equal proportion^2^. We generated ten subsampled datasets with this dataset that mixed two cell types at various proportions, with the rare cell type (Jurkat) proportions ranging from 0.5 to 5.0% (Methods). As CellSIUS required at least two major cell types in input, we therefore evaluated the four remaining methods with these datasets. GapClust and RaceID displayed superior performance than GiniClust and FiRE in all datasets, where GapClust performed slightly better than RaceID (Supplementary Fig. 7). FiRE produced improved predictions only when proportions of the rare cells were higher, consistent with the results reported by the authors of the FiRE algorithm^16^. GiniClust could not identify rare Jurkat cells in any of the datasets.

Inspecting the five methods on extreme conditions with only two rare cells (doublets) diluted in the major cell types with various proportions, we created a total of 140 datasets that mixed two CD14 + monocytes into two homogeneous populations of NK cells and B cells (0.08–5% of rare cell-type proportions, Methods). GapClust identified the two monocytes of all datasets, reflecting its sensitivity to rare cell type with extremely low frequencies (Supplementary Fig. 8). RaceID displayed a modest performance at a deficient proportion (0.08%) and a relatively high proportion of 5%. The efficacy of CellSIUS degraded as the rare cell proportion became lower. The remaining algorithms, including GiniClust and FiRE, failed to detect the two monocytes in all datasets. Taken together, these comparative analyses conclusively suggested that GapClust could provide superior performance in rare cell type detection.

Of note, the two-dimensional embeddings learnt by EDGE are represented with figures, which are not appropriate for direct comparison with the other five methods. Therefore, we have compared GapClust with EDGE by multiple simulation datasets with varying differential expression levels between the rare cell types and the abundant cell types, and different rates of dropout events (Supplementary Table 1). The results demonstrate that GapClust performs better than EDGE in cases with a low differential expression level (de.prob = 0.05, 0.1) between the rare cell type and major cell types and high rates of dropout events (0.6, 0.7 and 0.8) (Supplementary Figs. 9–13; Supplementary Table 2).

### GapClust is sensitive to cell type identity

**Fig. 3:**
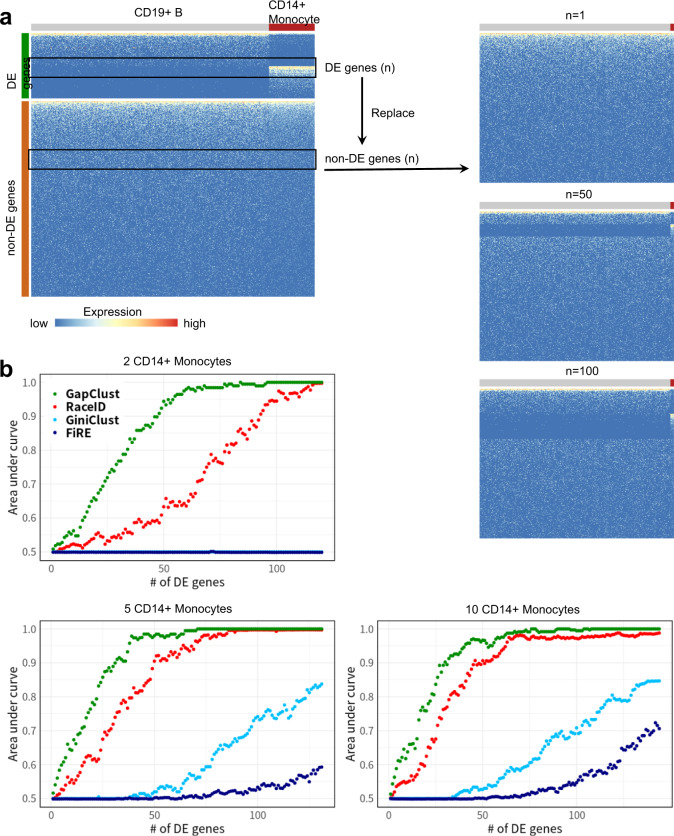
Benchmarking on the sensitivity of different approaches to cell-type identity. **a** Schematic overview of dataset perturbations. Starting from two cell types within the 68 k PBMC dataset (abundant CD19+ B cells and rare CD14+ monocytes), we firstly generated three datasets by subsampling 2, 5 and 10 monocytes and 500 B cells. Differentially expressed (DE) genes between two cell types were selected through a stringent criterion for each dataset, respectively. We replaced a fixed number of non-DE genes by the pre-identified DE genes. The count of replaced genes varied between 1 and the total number of DE genes. **b** The comparison of sensitivity of different methods to cell-type identity while varying the number of DE genes replaced.

To benchmark GapClust’s robustness and sensitivity with respect to the number of DE genes for varying incidence (i.e. the total number of rare cells), we subsampled three datasets comprising 500 CD19 + B cells and varying numbers of CD14 + monocytes (2, 5 and 10) from the 68 k PBMC dataset, respectively (Methods). The rare monocytes represented about 0.4%, 1% and 2% of the total population, respectively. A total of 120, 131 and 144 DE genes between two cell types were identified with stringent criteria for each dataset (Methods). Given a dataset, for each iteration of the experiments, we replaced a fixed number of non-DE genes with an equal number of DE genes, and the count of replaced genes varied between 1 and the total number of DE genes to evaluate the sensitivity of GapClust in detecting the rare cell type (Fig. 3a). We repeated the aforementioned procedure 100 times to obtain the average area under the curve (AUC) of receiver operating characteristics in minor population detection for each count of DE genes.

For comparison, we also applied GiniClust, RaceID and FiRE in each experimental replicate. Similarly, CellSIUS was not applicable due to only one major cell type. All four methods struggled to detect the monocytes with only a few DE genes (Fig. 3b). However, the performance of GapClust improved more sharply than competitors when more DE genes were introduced. In the setting with two CD14 + monocytes, when 50 DE genes were present, GapClust offered an average AUC of 0.89, far higher than the performances of RaceID, GiniClust and FiRE, which were 0.63, 0.5 and 0.5, respectively (Fig. 3b). We consistently observed similar results in the other two scenarios with 5 or 10 monocytes, which demonstrated superior sensitivity of GapClust compared with existing approaches.

### GapClust is scalable and fast

**Fig. 4:**
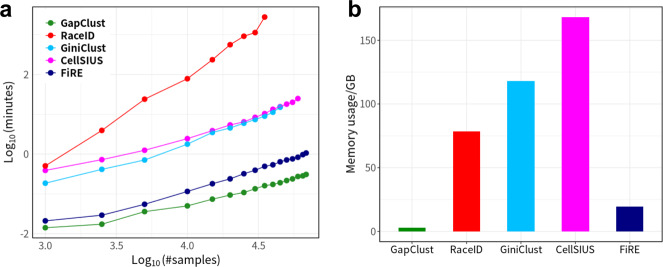
GapClust is fast and memory efficient. **a** Execution time recorded for the five methods (GapClust, RaceID, GiniClust, CellSIUS and FiRE) while varying the number of cells from 1 k to ~68 k. **b** Maximum memory usage for the five methods in processing cells with number varying from 1 k to ~68 k.

With the rapidly increasing throughput of single-cell technology, up to millions of transcriptomes can now be profiled at single-cell resolution. The scalability of computational algorithms in recent years has become a major concern for scRNA-seq data analysis. To evaluate the computational efficiency of RaceID, GiniClust, CellSIUS, FiRE and GapClust, we tracked the time taken by these methods with varying input data sizes on a single machine with forty cores of 2.5 GHz Intel Xeon CPU and 183.59 GB of memory. For comparison, we subsampled 16 datasets from the 68 k PBMC dataset, comprising between 1000 and ~68,000 expression profiles. Consistent with the previous study, GiniClust encountered a runtime error when the input data consisted of beyond ~45,000 cells^16^ (Fig. 4a, and Supplementary Table 3). RaceID was computationally expensive compared to other methods, taking the most prolonged duration to run, with just over 5000 cells. By contrast, GapClust and FiRE had processed expression profiles of ~68,000 cells in seconds, and GapClust was three times faster than FiRE (Supplementary Table 3).

Demanding memory usage posed another challenge to the scalability of the computational algorithm in processing large-scale single cell transcriptomics. Previous studies focused on comparing the computation efficiency among rare cell detection algorithms without addressing memory issue^14,16^. We compared the maximum memory utilisation of each algorithm when processing the 16 datasets above. CellSIUS consumed the most memory resource (over ~150 GB) among the five methods (Fig. 4b, and Supplementary Table 4). The memory usages of GiniClust and RaceID were 118 GB and 78 GB, respectively. It should be noted that these three methods processed 14, 11 and 9 of the 16 datasets, with the largest one consisting of 60,000, 45,000 and 35,000 expression profiles. FiRE achieved drastic improvements in memory utilisation (<20 GB) compared with the above three methods. GapClust cut down the memory requirement further (*~*3 GB) when handling the same datasets. Taken together, GapClust could be scalable to huge scRNA-seq datasets with a higher speed and much lower memory demand, making it more user friendly.

### GapClust identifies rare tuft cells in intestine crypt

Tuft cells are rare, chemosensory epithelial cells that were identified over 60 years ago^24,25^. These rare cells did not generate interest until three reports in 2016 identified tuft cells to be central in type 2 immune circuits and crypt epithelial cell progenitors^26–28^. A recent study by Arshad et al.^29^ delineated multiple sub-types of enriched crypts by analysing the expression profiles of fluorescence-activated cell sorting sorted cells from crypt zone. The authors observed that tuft cells, enterocytes, enteroendocrine cells and some other sub-types were increased in numbers following irradiation.

**Fig. 5:**
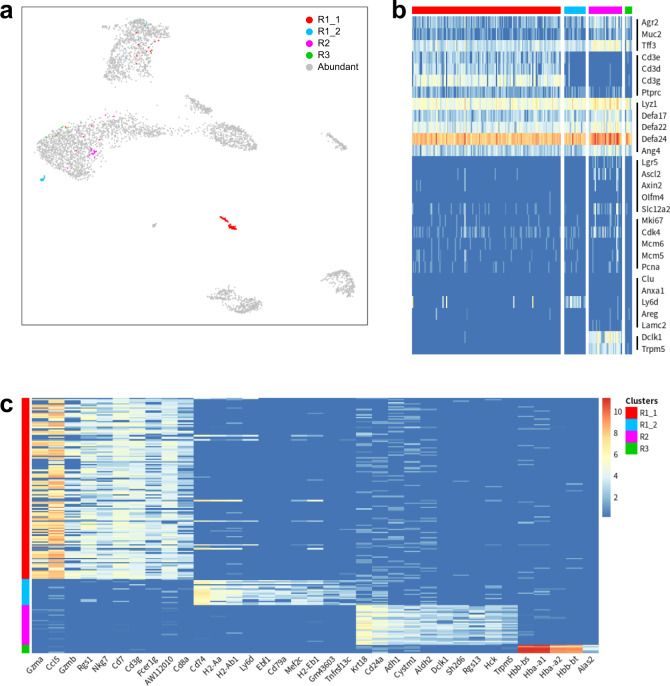
Analysis of the intestine dataset. **a** The two-dimensional embeddings learnt by EDGE on the intestine dataset with rare cell types labeled in different colours. **b** Expression of differentiated lineage marker genes across all rare cell types. Note that marker genes (*Dclk1*, *Trpm5*) of tuft cells reported by Arshad et al.^29^ are enriched in cluster R2. **c** Heatmap of top differentially expressed genes for each minor cluster. The same colour-coding scheme is used in all panels.

We asked whether tuft cells could also be detected in enriched crypts prior to irradiation. To this end, we applied GapClust to the expression profiles of 4491 non-irradiated cells published by Arshad et al.^29^. GapClust reported a total of three rare cell types, and cluster 1 was divided into two sub-populations (R1_1, R1_2) (Fig. 5a). With the marker genes (*Dclk1*, *Trpm5*) reported by Arshad et al.^29^, we easily identified that the 29 cells from cluster R2 were tuft cells (Fig. 5b). For these four groups, we conducted differential expression analysis to filter cell-type specific genes, which clearly distinguished each cluster from the remaining cell types (Fig. 5c). Marker genes such as *Cd8a*, *Cd3g*, *Ccl5*, *Gzma* and *Gzmb*, were specifically expressed in sub-population R1_1, suggesting that these 131 cells belonged to intestine-infiltrating CD3 + CD8 + T cells^30^. Consequently, the 19 cells from the remaining sub-population (R1_2) would be implicated with intestinal immunity. Furthermore, high expression of antigen presentation genes (major histocompatibility complex (MHC) class II [*H2-Aa*, *H2-Ab1*, *H2-Eb1*], *Cd74*) confidently demonstrated the functionality of these rare cells^31^. A total of six cells in the smallest cluster R3 presented multiple erythrocyte markers, including *Alas2* and hemoglobin genes (*Hbb-bs*, *Hba-a1* and *Hbb-bt*), implying the pollution of erythrocytes during cell sorting^32^.

### GapClust identifies rare CD14 + monocytes in large 68 k PBMC dataset

**Fig. 6:**
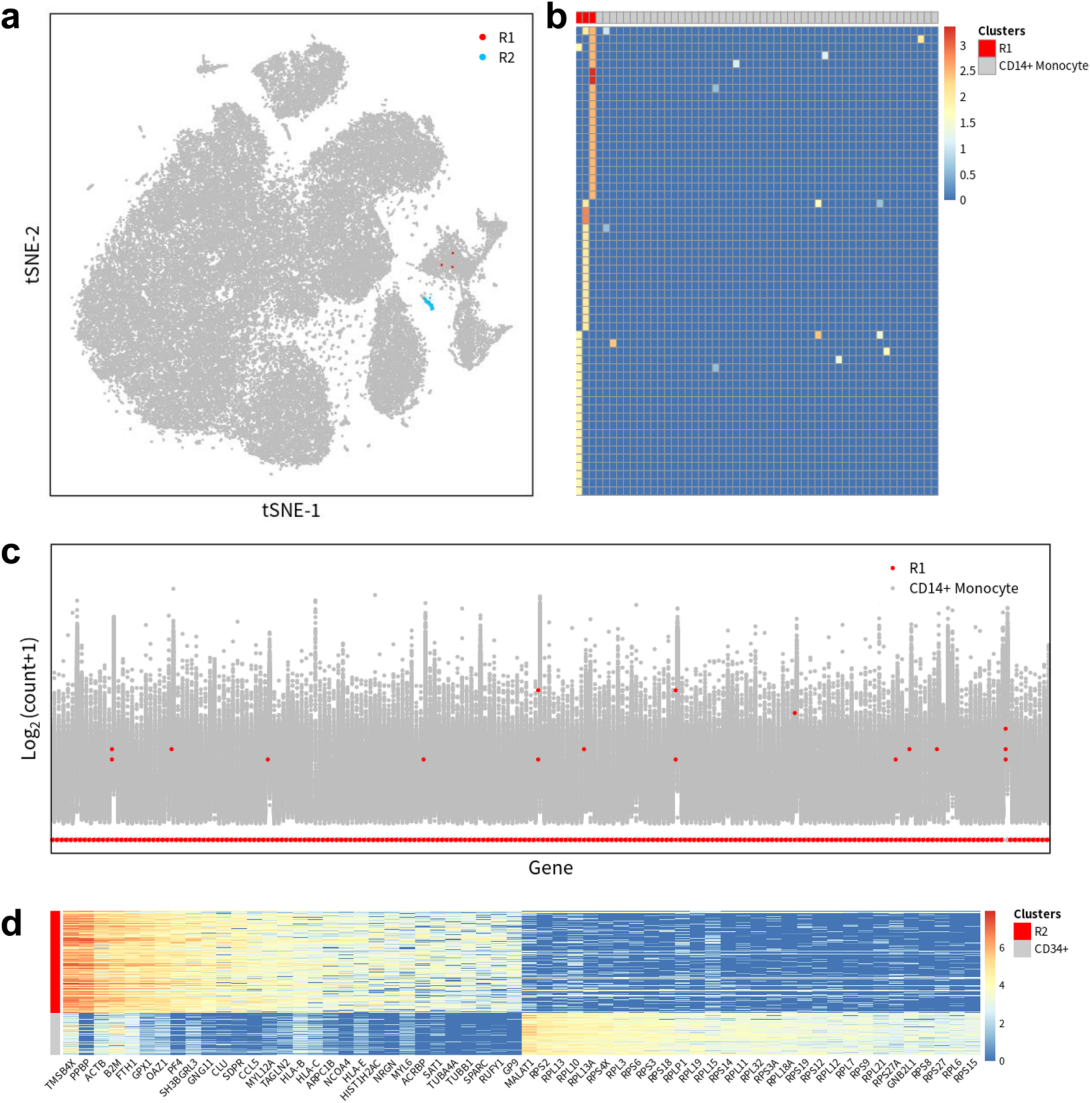
Identification of novel, rare cell types from the 68 k PBMC dataset. **a** The tSNE plot of the full 68 k PBMC dataset with rare cell types labeled in different colours. **b** Heatmap of the top differentially expressed genes between minor cluster R1 and the remaining CD14+ monocytes. **c** Expression distribution of 218 genes with absolute log_2_ fold-change larger than 1 between the minor cluster R1 and the remaining CD14+ monocytes. **d** Heatmap of the top differentially expressed genes between minor cluster R2 and the remaining CD34+ cell population.

The well-studied 68 k PBMC dataset was extensively adopted by various computation algorithms as the benchmark for scalability to large datasets. Both GiniClust and FiRE were applied to analyse these PBMCs and identified the rare cell type of (CD34 + ) megakaryocytes^14,16^. Our interest was whether GapClust could replicate the findings and discover unreported rare cell types. Within the full 68 k PBMC dataset, GapClust identified two rare cell types (Fig. 6a). Surprisingly, the three cells of the first group R1 were only captured by GapClust, but not by GiniClust and FiRE. We found that the three cells were classified as CD14 + monocytes with the established labels and explored the DE genes between these cells and the remaining CD14 + monocytes. Though up-regulated compared with remaining monocytes, the 57 DE genes did not show any co-expression pattern within the three cells, making it difficult for these cells to form a cluster (Fig. 6b). So, we examined the remaining genes with absolute log_2_ fold-change larger than 1 between two groups and found that the majority of 218 down-regulated genes were not expressed in all three cells from cluster R1 in contrast with the remaining CD14 + monocytes (Fig. 6c). These unexpressed genes included lymphocyte markers such as *CD4*, *CD52*, *CD68*, *CD74*, *CD97*^33–35^, and MHC class genes (*HLA-C*, *HLA-DMA*, *HLA-DQA1*, *HLA-DQA2*, *HLA-DQB1*, *HLA-E*^36^), suggesting that these three cells were different from the remaining CD14 + monocytes. Additional validation would be necessary to determine whether this cluster was functionally distinct.

Within the second rare cell type R2, GapClust detected 185 CD34 + megakaryocytes successfully, like GiniClust and FiRE. We observed that the remaining 77 CD34 + cells within PBMCs were not included and thus conducted differential expression analysis between cluster R2 and the remaining CD34 + cell population, with a total of 3339 DE genes identified (Fig. 6d). Among these genes, MHC class genes such as *HLA-B*, *HLA-C*, *HLA-E* and inflammatory chemokine gene *CCL5* were highly expressed in the cells of cluster R2. In contrast, hematopoietic differentiation regulating lncRNA *MALAT1*^37^ and various ribosomal protein genes (*RPS2*, *RPL13*, *RPL10*, *RPL13A*) were up-regulated in the remaining CD34 + cells, implying a distinct subset of megakaryocytes. To summarise, GapClust could not only recover the rare CD34 + cells, but also identify rare cell type of only three cells (~0.004%) classified as CD14 + monocytes in the large 68 k PBMC dataset.

## Discussion

Computational methods including GiniClust^14^, RaceID^13^, CellSIUS^15^ and FiRE^16^ have made rare cell type detection feasible by various innovative design. However, the balance between accuracy and scalability has been the primary concern of all times. In terms of accuracy, GapClust consistently performs better than existing approaches in extensive simulation experiments, including five scenarios of simulated datasets with varying numbers of rare cells, different levels of differential expression between the rare cell type and other cell types by Splatter, 99 subsampled datasets from the 68 k PBMC dataset, ten subsampled datasets from the 293T-Jurkat dataset and 140 subsampled datasets from the 68 k PBMC dataset for doublets identification. In sensitivity analyses, GapClust offers higher prediction accuracy with the same DE genes between the rare cell type and the abundant cell type than GiniClust, RaceID and FiRE in all experiments. In terms of scalability to the large datasets subsampled from the 68 k PBMC dataset, GapClust utilises far less computational time and memory resources than GiniClust, RaceID and CellSIUS, and is slightly more efficient than FiRE. Comparisons between EDGE and GapClust with multiple simulation experiments demonstrate that GapClust performs better in cases with a low level of differential expression between the rare cell type and major cell types and high rate of dropout events.

In addition, GapClust has been successfully applied to multiple real datasets. Within the full 68 k PBMC dataset, GapClust identifies a new minor cluster of only three rare cells classified as CD14 + monocytes, which GiniClust and FiRE have not reported^14,16^. Tuft cells are identified in the intestinal dataset, validated by existing maker genes including *Dclk1* and *Trpm5*. Moreover, we have identified rare cell types in the heterogenous human hippocampus scRNA-seq dataset^38^ (Supplementary Fig. 14), the *Drosophila* wing disc datasets^39^ (Supplementary Figs. 15, 16), the mouse embryonic stem cells dataset^40^ (Supplementary Fig. 17), the mouse somatosensory cortex dataset^41^ (Supplementary Fig. 18; Supplementary Table 5), the Jurkat dataset^16^ (Supplementary Fig. 19), the *Bacillus subtilis* cells dataset^42^ (Supplementary Fig. 20) and the mouse tracheal epithelial cells dataset^43^ (Supplementary Fig. 21). The same rare cell type identified in the four *Drosophila* wing disc datasets of two scenarios with replicates strongly demonstrates that GapClust can recover rare cell types from different batches of datasets.

Intuitively, GapClust takes advantage of the gap information between the minor cluster and its neighbouring cluster to allow the rare cells within the minor cluster to stand out by designing the second-derivative (reflecting the variation of functions) like statistic ∆∆*D*_*<m, k>*_. Meanwhile, unlike most of the competitors struggling to search for rare cell informative genes, GapClust learns the cluster size and rare cells using simple arithmetic calculation, illustrating the high efficiency and scalability to large datasets. Of note, GapClust does not report all rare cell types of the same size on a single run but identifies the rare cell type with the largest gap between its neighbouring cluster and itself. To discover these hidden clusters, users can rerun GapClust in no time with the identified rare cell types removed. Moreover, with the simulation datasets by Splatter, we have evaluated the performance of GapClust based on Euclidean and Manhattan distance, respectively, and found that Euclidean distance is the optimal choice for GapClust, due to its robust performance and fast calculation (Supplementary Fig. 22).

In principle, GapClust does not apply to the occasional situation where only the rare cell-type-specific genes distinguish the rare cell population from the abundant cells, as these features cannot be captured by vst feature selection in Seurat due to low variance. In practice, except for the rare cell-type-specific genes, other up-regulated genes, which are expressed in the majority of the whole cell population and especially highly expressed in the rare cells, would have high variances. Furthermore, the down-regulated genes low expressed in the rare cells and highly expressed in remaining abundant cells, would have high variances and also be found by vst method.

GapClust is currently the fastest rare-cell detection method to analyse such large datasets with less memory resources, as demonstrated by the benchmark using the 68 k PBMC dataset. Such unrivalled efficiency, combined with high accuracy and sensitivity in rare cell detection, allows GapClust to be utilised as a fundamental tool for new cell type discovery within ultra-large scRNA-seq datasets.

## Methods

### Data preprocessing

The processed 68 k PBMC dataset was represented as UMI counts. Genes that were expressed in less than three cells were excluded, leaving 20,387 genes for further analysis, and cells expressing <200 genes were also excluded. A total of 68,579 cells passed this filter. The normalisation procedure was accomplished using the scran package^44^. Cell type labels were determined based on the maximum correlation between the gene-expression profile of each cell to 11 purified cell populations, using the code provided by 10× Genomics.

The processed 293T-Jurkat dataset had been filtered and normalised with the scran package. Moreover, the intestine dataset was represented as UMI counts. Genes that were expressed in less than three cells were excluded, leaving 16,091 genes for further analysis. Cells expressing <200 genes were excluded. A total of 4491 cells remained for further analysis. Filtered data was subjected to global median normalisation, as the size factors by the scran package had negative values.

For the remaining datasets, genes that are expressed in at least two cells were retained for downstream analysis. Each scRNA-seq dataset was normalised using median normalisation. The normalised matrix was then log_2_ transformed after the addition of 1 as a pseudo count.

### GapClust method details

The GapClust pipeline includes the following steps.

1. Obtaining *K* nearest neighbours for all cells: For each gene in the input expression matrix, the variance is calculated with the vst feature selection method in Seurat package^5^. Based on the distribution of variance values, top genes can be filtered by the elbow point of the density plot. Principal component analysis (PCA) is applied to the expression matrix for dimension reduction using the irlba package. With the top 50 principal components, the *K* nearest neighbours of all cells are obtained using the rflann package.
2. Identification of candidate *k*: Let *D*_*<m, k>*_ denote the distance between cell *m* and its *k*th neighbour,

1 $$\begin{array}{c}{D}_{ < {{m}},\,{{k}} > }=\sqrt{\mathop{\sum }\limits_{i=1}^{50}{({x}_{ < {{m}},\,{{i}} > }-{x}_{ < {{k}},\,{{i}} > })}^{2},}\end{array}$$

where *x*_*<m, i>*_ is the *i*th principal component by PCA in cell *m*, *x*_*<k, i>*_ is the *i*th principal component of cell *m*’s *k*th nearest neighbour and ∆*D*_*<m, k>*_ denote the first-order derivative of *D*_*<m, k>*_ with respect to *k*. Since *k* is discrete and increases by 1, for each cell *m*, ∆*D*_*<m, k>*_ can be obtained by calculating the difference of *D*_*<m, k>*_ and *D*_*<m, k−1>*_.

2 $$\begin{array}{c}\Delta {D}_{ < {{m}},\,{{k}} > }={D}_{ < {{m}},\,{{k}} > }-{D}_{ < {{m}},\,{{k}}-1 > }.\end{array}$$

Furthermore, the second-order derivative like statistic ∆∆*D*_*<m, k>*_, which measures the total difference between ∆*D*_*<m, k>*_ and each of ∆*D*_*<m, k−1>*_, ∆*D*_*<m, k+1>*_, can be calculated according to the formula below:

3 $$\Delta \Delta \begin{array}{c}{D}_{ < {{m}},\,{{k}} > }=\Delta {D}_{ < {{m}},\,{{k}} > }-\Delta {D}_{ < {{m}},\,k-1 > }+\Delta {D}_{ < {{m}},\,{{k}} > }-\Delta {D}_{ < {{m}},\,k\,+\,1 > }.\end{array}$$

For each *k* between 2 and *K*−1, the ∆∆*D*_*<m, k>*_ of each cell (*m* = 1, …, *N*) is stabilised by taking the average of the ∆∆*D*_*<m, k>*_ values of its nearest neighbour and itself. With the top *k* values removed, the remaining ∆∆*D*_*<m, k>*_ values are kept as abundant cells if it is ≥q1 + 1.5 × IQR and ≤q3 + 1.5 × IQR, where q1, q3 and IQR denote the first quartile, third quartile and the interquartile range (75th percentile –25th percentile), respectively. As to hypothetic rare cells, the ∆∆*D*_*<m, k>*_ values of the top cell and its *k*−1 nearest neighbours are averaged to represent the robust ∆∆*D*_*<m, k>*_ value. Then the filtered ∆∆*D*_*<m, k>*_ values representing abundant cells and two averaged ∆∆*D*_*<m, k>*_ values representing hypothetic rare cells are combined and subject to skewness calculation using the e1071 package. Here, the added number of averaged ∆∆*D*_*<m, k>*_ value is set as two rather than *k* to maintain sensitivity for relatively large minor clusters, as skewness of ∆∆*D*_*<m, k>*_ tends to shrink with increasing *k*. Candidate *k* can be identified if the skewness_*<k>*_ > 2.

Identification of rare cell types: For each candidate *k*, we find the cell with the largest ∆∆*D*_*<m, k>*_ value among *N* cells and its *k*−1 nearest neighbours and allocate skewness_*<k>*_ to these *k* cells. We find the largest skewness value for each cell with skewness values and allocate the corresponding *k* to this cell. In the last step, the cells labeled with *k* whose count is equal to *k* are determined as a rare cell type.

### Simulation studies

We utilised the R package Splatter to simulate scRNA-seq datasets for comparison between the five methods. We first simulated scRNA-seq data with 1200 cells and 5000 genes, which comprises three cell types with a ratio of 10:45:45. Secondly, varying numbers of cells between 2 and 100 were randomly sampled from the cell type 1 as the rare cell type, and 500 cells were randomly sampled from the other two cell types as major cell types. For the scenarios with different differential expression levels, datasets comprising 1000 cells were simulated with the ratio of two cell types 1:99, three cells were randomly sampled from the cell type 1 as the rare cell type, and 500 cells were randomly sampled from the cell type 2 as the major cell type. The differential expression level was determined by varying the de.prob parameter (0.1, 0.2, 0.4, 0.6 and 0.8) in Splatter. For the scenarios with different rates of dropout events, the rate of dropout events was determined by varying the dropout.mid parameter (0.1068, 0.2033, 0.3090, 0.4050, 0.5023, 0.6078, 0.7041, 0.8026 and 0.9067) in Splatter.

### EDGE visualisation

Dimension reduction by EDGE was performed using the EDGE R package^18^. The EDGE algorithm was run using the (log_2_(normalised counts + 1) matrix to obtain a separate two-dimensional projection.

### tSNE visualisation

Dimension reduction by tSNE was performed using the Rtsne package^45^. The tSNE algorithm was run using the (log_2_(normalised counts + 1) matrix to obtain a separate two-dimensional projection.

### *F*_1_ score computation for the simulation study

*F*_1_ score reflects the detectability of rare cells by the harmonic mean of precision and recall in a two-class experiment, which can be obtained from the confusion matrix. The *F*_1_ score is then computed as follows:

4 $$\begin{array}{c}{F}_{1}\,score=2\times \frac{{\rm{precision}}\times {\rm{recall}}}{{\rm{precision}}+{\rm{recall}}}.\end{array}$$

### Simulation to assess sensitivity to DE genes

To evaluate the sensitivity of GapClust to cell-type identity, we generated a synthetic scRNA-seq data comprising two cell types based on the 68 k PBMC dataset. To ensure homogeneity, we started with 96 CD14+ monocytes and 2686 CD19+ B cells from the largest cluster of each cell type by DBSCAN^46^, and sampled 500 CD19+ B cells randomly as the major cell type. We kept genes whose expression counts exceeded 2 in at least three cells for analysis. Differentially expressed (DE) genes were filtered using the Wilcoxon’s rank-sum test with a false discovery rate (FDR) adjusted *p* value cutoff of 0.05 and inter-group absolute fold-change cutoff of 1. With 80 up-regulated and 1002 down-regulated genes identified, we removed the top 80 down-regulated genes by the absolute fold-change and all 80 up-regulated genes from the data and kept it as a separate set. A total of 5009 genes with a *p* value > 0.05 were kept as a separate set of non-DE genes. On the filtered datasets, we randomly sampled 2, 5 and 10 CD14+ monocytes and combined them with the 500 CD19+ B cells to generate three scenarios to benchmark the sensitivity of GapClust to the number of DE genes.

### Differential expression analysis

Traditional Wilcoxon’s rank-sum tests were applied to identify DE genes with an FDR adjusted *p* value < 0.05 and an inter-group absolute log_2_ fold-change > 1. For a given gene, the fold-change value was measured between group-wise mean expression values.

### Identification of rare cell types with GiniClust, RaceID, CellSIUS, FiRE and EDGE

GiniClust2 package was obtained from GitHub (dtsoucas/GiniClust2, version as of 14 Jul 2018). The analysis was run with default parameters: MinPts = 3, eps = 0.45, *k* = 10 for all datasets, except that MinPts was adjusted to 2 in case of doublets identification, whilst other parameters were set to their defaults.

RaceID package was directly applied to the normalised expression matrix, with all parameters at their default values, except that the initial clusters were determined according to abundant cell types rather than by *k*-medoids.

CellSIUS package was downloaded from GitHub (Novartis/CellSIUS, version as of 3 Jun 2019). The initial major cell types were determined using *k*-means with a data-specific *k*. Other parameters were set to their defaults, except for the min_n_cells parameter, which was set as 2 in the case of detecting any doublets.

FiRE package was obtained from GitHub (princethewinner/FiRE, version as of 9 Aug 2019). All parameters were set to their defaults. As to IQR thresholding criteria for rare cell detection, we also tried 1.0 and 0.5 for the IQR coefficient.

EDGE package was obtained from GitHub (shawnstat/EDGE, version 1.0). All parameters were set to their defaults.

### The 68 k PBMC data subsampling

The full 68 k PBMC dataset was downsampled for model evaluation. We considered only three cell types whose transcription profiles were distinct from one another (Fig. 2a), including CD19+ B cells, CD14+ monocytes and CD56+ NK cells, which were defined in the same way as in Daphne et al.^14^. Based on these three cell types, we created a total of 99 subsampled datasets in the following manner: 2–100 CD14+ monocytes were randomly sampled from the monocyte population to form the rare cell group for the 99 datasets, respectively. Then, for each dataset, 500 cells were randomly sampled from the CD56+ NK and CD19+ B cells to form the two abundant cell types.

As to the evaluation of doublets detection, we created seven sets of 20 subsampled datasets, each based on the three cell types above in the following manner: two CD14+ monocytes were randomly sampled from the monocyte population to form a rare cell group for the 140 datasets. Then for each set of 20 datasets, cells were randomly sampled from the NK and B cells in specified number (NK cell: 13, 25, 50, 100, 200, 400, 800; B cell: 25, 50, 100, 200, 400, 800, 1,600) to form the abundant cell types.

### The 293T-Jurkat data subsampling

Within a total of ~3200 cells, the authors of the study determined the cell types by bioinformatically exploiting SNV profiles of these cells^2^. We created ten subsampled datasets in the same manner as Aashi et al.^16^: 1540 cells were randomly selected from 293T cell population to form the common cell cluster. Then various numbers (8, 16, 24, 32, 40, 48, 56, 65, 73 and 82) of cells were randomly sampled from Jurkat population for ten datasets with the proportion of Jurkat cells varying between 0.5 and 5%.

### Reporting summary

Further information on research design is available in the Nature Research Reporting Summary linked to this article.

## Supplementary information

Supplementary Information

Reporting Summary

## Data Availability

The study uses multiple publicly available scRNA-seq datasets. Both 68 k PBMC and 293T-Jurkat cell datasets are available from https://support.10xgenomics.com/single-cell-gene-expression/datasets. The intestine dataset can be assessed at the GEO under accession code GSE123516. Moreover, the accession codes of other datasets are: the human hippocampus scRNA-seq dataset (GSE131258), the *Drosophila* wing disc datasets (GSE155543), the mouse embryonic stem cells dataset (GSE65525), the mouse somatosensory cortex dataset (GSE60361), the Jurkat dataset (GitHub: princethewinner/FiRE/data), the *Bacillus subtilis* cells dataset (GSE151940) and the mouse tracheal epithelial cells dataset (GSE103354).

## Code availability

GapClust is implemented in R and the source code has been deposited at https://github.com/fabotao/GapClust and Zenodo (10.5281/zenodo.4765514)^47^.
