# Supplementary material for: GapClust is a light-weight approach distinguishing rare cells from voluminous single cell expression profiles
Source: Nat Commun. 2021 Jul 7;12:4197. doi: 10.1038/s41467-021-24489-8 (PMC8263561; doi:10.1038/s41467-021-24489-8)
Supplement: Supplementary file 1 — Supplementary Information [file 41467_2021_24489_MOESM1_ESM.pdf]

GapClust is a light-weight approach distinguishing rare cells from voluminous single cell expression profiles

Supplementary Material

Fa et al.

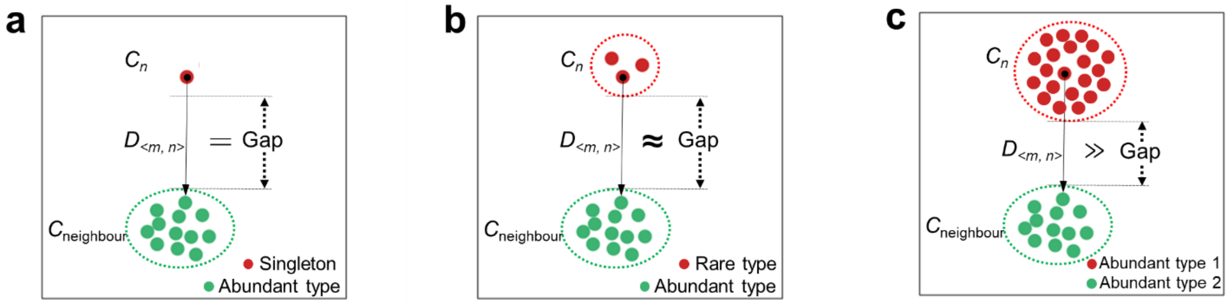

Supplementary Figure 1 Three scenarios of the gap between two clusters. **a** There is only one cell in C_n. **b** There are a few cells in rare cell type C_n. **c** There are many cells in the abundant cell type C_n. $D_{<m, n>}$ denotes the distance between each cell m in C_n and its nth neighbour, which is the nearest neighbour of cell m in $C_{\text{neighbour}}$. The clusters in different colours denote different cell types. In scenario **a** where there is only 1 cell (singleton) in C_n, the gap can be directly obtained by calculating the distance $D_{<m, n>}$ of the single cell m in C_n to its nearest neighbour cell in $C_{\text{neighbour}}$; In scenario **b**, the rare cell type C_n comprises a few cells, although the gap cannot be directly obtained, we can still approximate the gap by calculating the distance $D_{<m, n>}$ of any given cell m in C_n to its nearest neighbour cell in $C_{\text{neighbour}}$; In scenario **c**, there are many cells in the abundant cell type C_n, we cannot approximate the gap like the above two scenarios again, as $D_{<m, n>}$ is far larger than the gap for most of cells in C_n. Therefore, in the scenario **b** of rare cell type, the gap can be approximated by calculating the distance $D_{<m, n>}$ of any given cell in C_n to its nearest neighbour cell in $C_{\text{neighbour}}$.

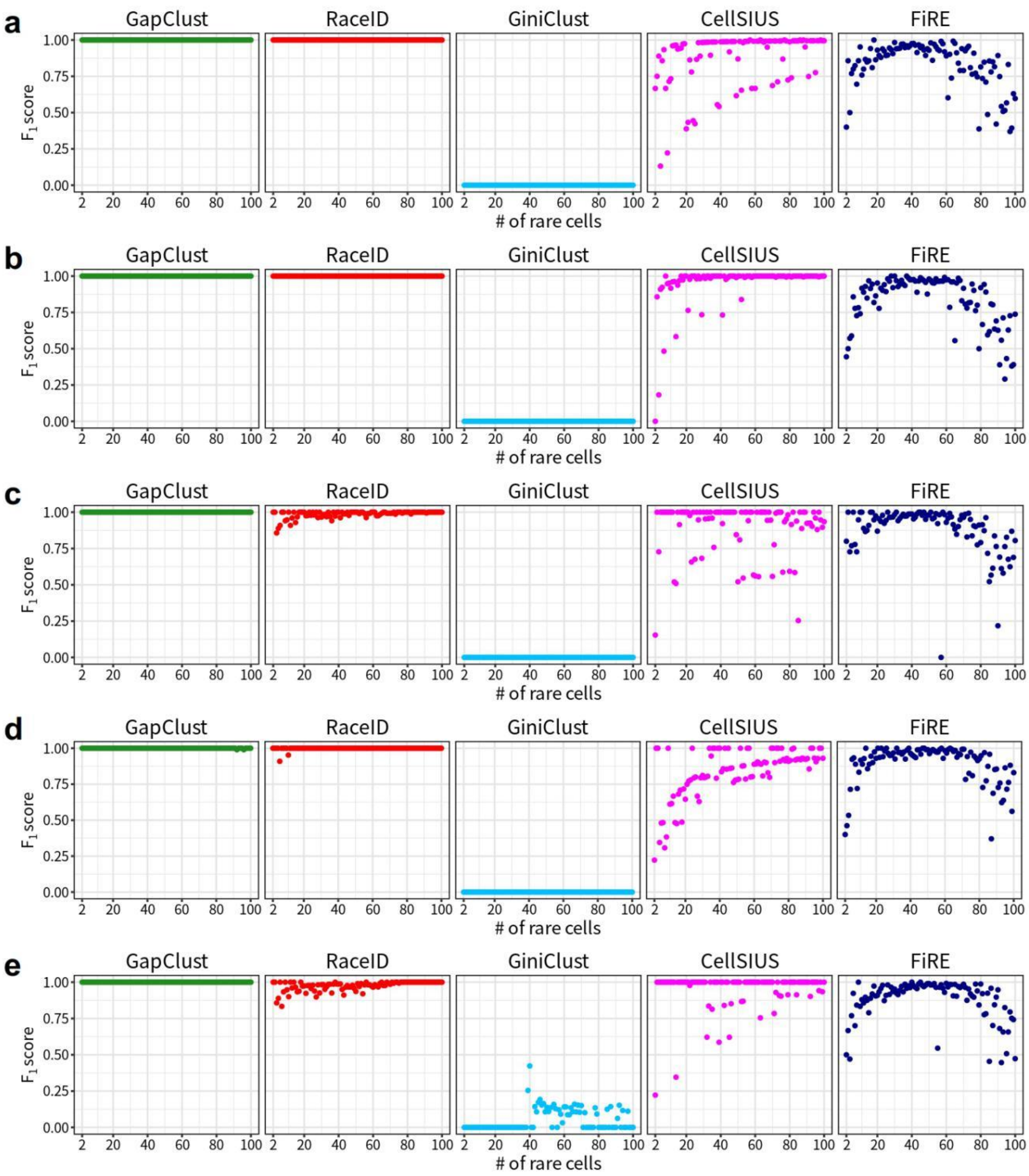

Supplementary Figure 2 The performance comparison of GapClust, RaceID[1], GiniClust[2], CellSIUS[3] and FiRE[4] in simulation datasets by Splatter[5]. The results (**a**-**e**) represent different levels of differential expression between the rare cell type and major cell type using different de.prob parameters in Splatter.

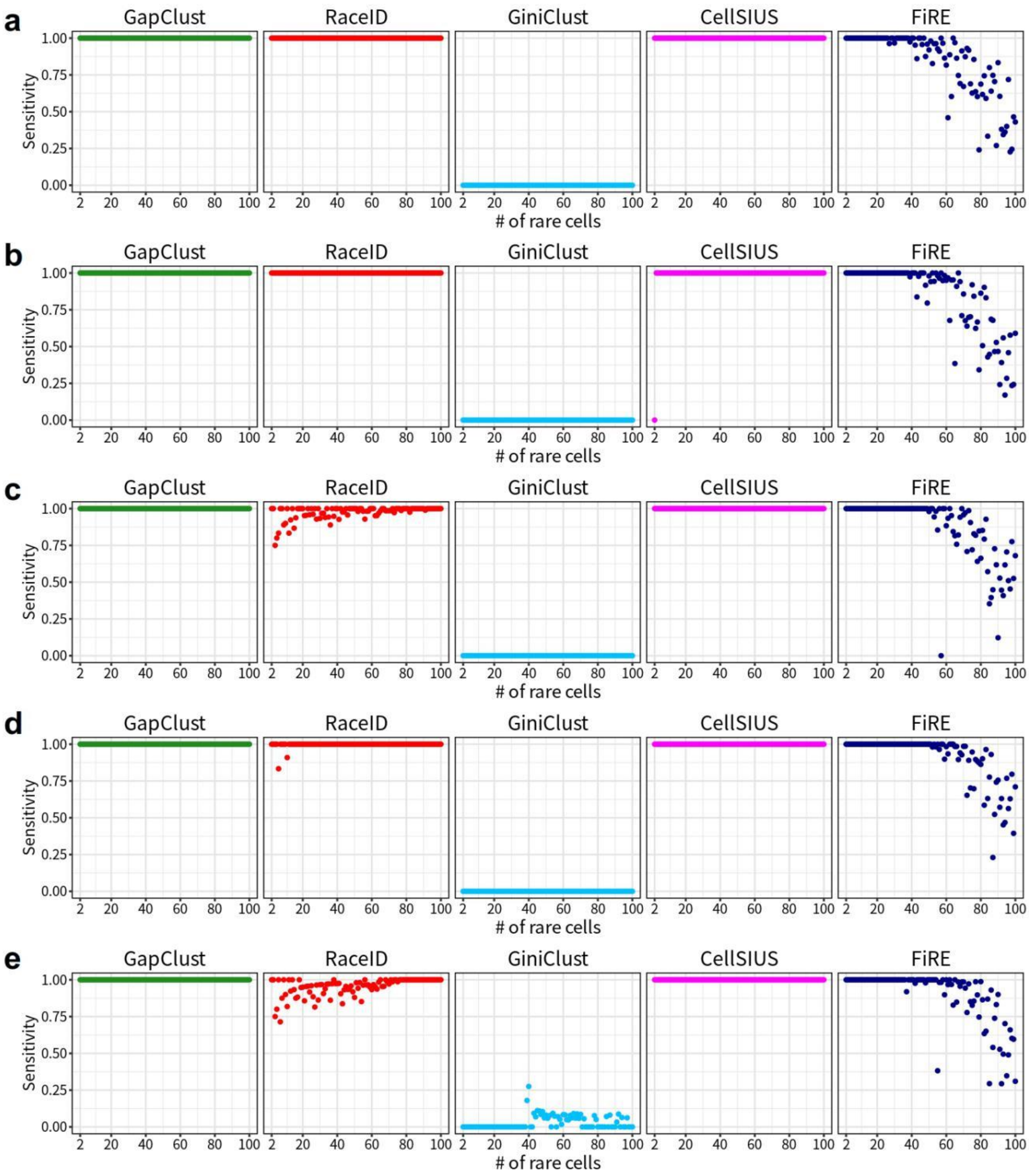

Supplementary Figure 3 Sensitivity comparison of GapClust to RaceID, GiniClust, CellSIUS and FiRE for varying incidence of the rare cell type in the simulated datasets by Splatter. Here we generate datasets comprising two abundant cell types of 500 cells and from 2 up to 100 rare cells. The five subplots (**a-e**) represent the results from the datasets with different levels of differential expression between the rare cell type and the major cell type by varying the de.prob parameter of Splatter: **a** de.prob=0.1, **b** de.prob=0.2, **c** de.prob=0.4, **d** de.prob=0.6, **e** de.prob=0.8.

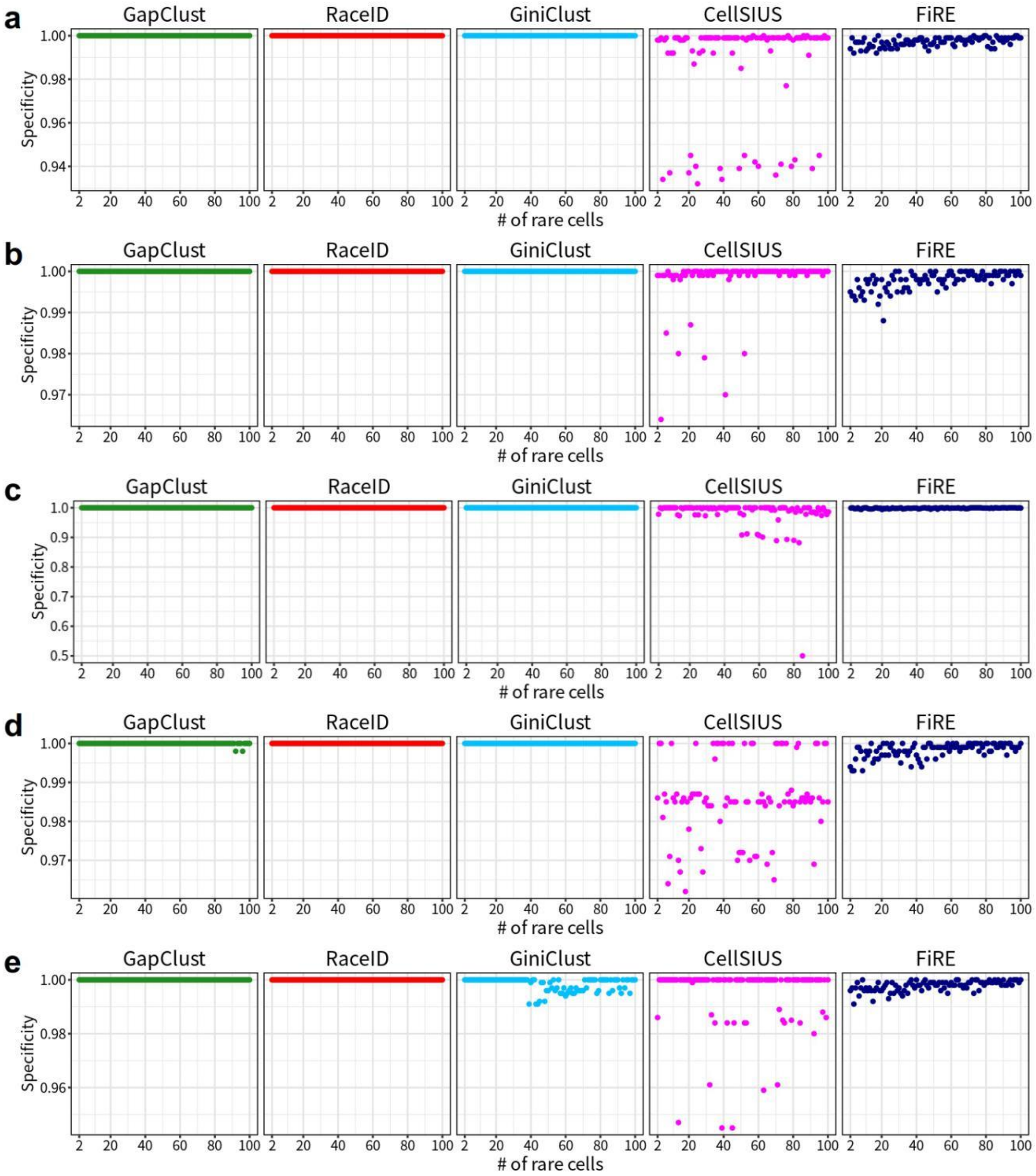

Supplementary Figure 4 Specificity comparison of GapClust to RaceID, GiniClust, CellSIUS and FiRE for varying incidence of the rare cell type in the simulated datasets by Splatter. Here we generate datasets comprising two abundant cell types of 500 cells and from 2 up to 100 rare cells. The five subplots (**a-e**) represent the results from the datasets with different levels of differential expression between the rare cell type and the major cell type by varying the de.prob parameter of Splatter: **a** de.prob=0.1, **b** de.prob=0.2, **c** de.prob=0.4, **d** de.prob=0.6, **e** de.prob=0.8.

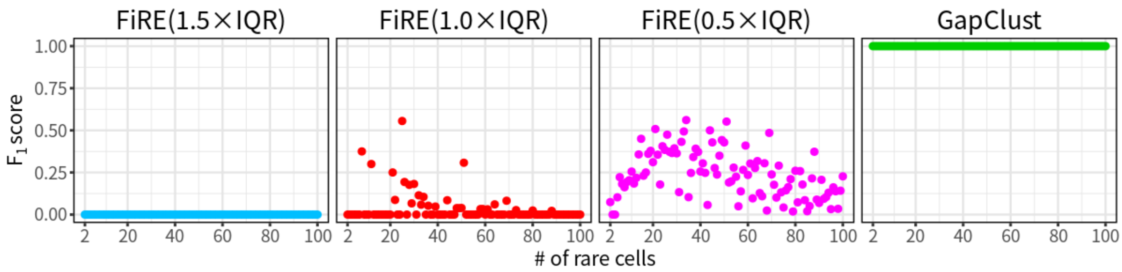

Supplementary Figure 5 Performance comparison of GapClust to FiRE for rare cell detection in simulated datasets subsampled from the 68 k PBMC dataset, quantified by F_1 score. FiRE method is applied with three different IQR-thresholding-criteria including q3 + 1.5 × IQR, q3 + 1.0 × IQR and q3 + 0.5 × IQR, where q3 and IQR denote the third quartile and the interquartile range (75th percentile - 25th percentile), respectively, of the number of FiRE scores across all cells.

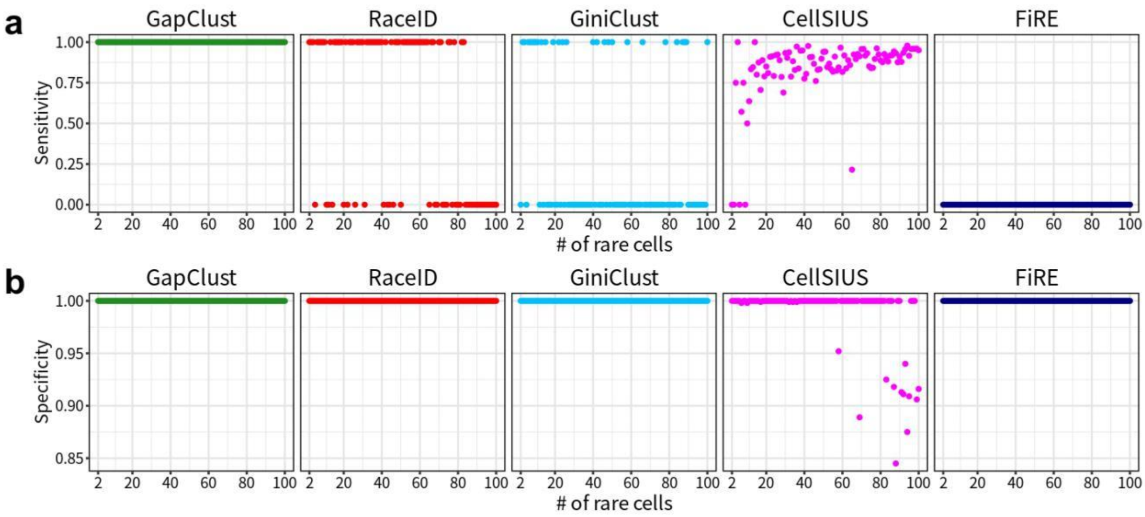

Supplementary Figure 6 Comparison of GapClust to RaceID, GiniClust, CellSIUS and FiRE for varying incidence of the rare cell type in the 99 subsampled datasets from the 68 k PBMC dataset. Here we use 500 CD19+ B cells as type 1, 500 CD56+ NK cells as type 2, and between 2 and 100 CD14+ monocytes as the rare cell type. **a** Sensitivity comparison among the five approaches, **b** Specificity comparison among the five approaches.

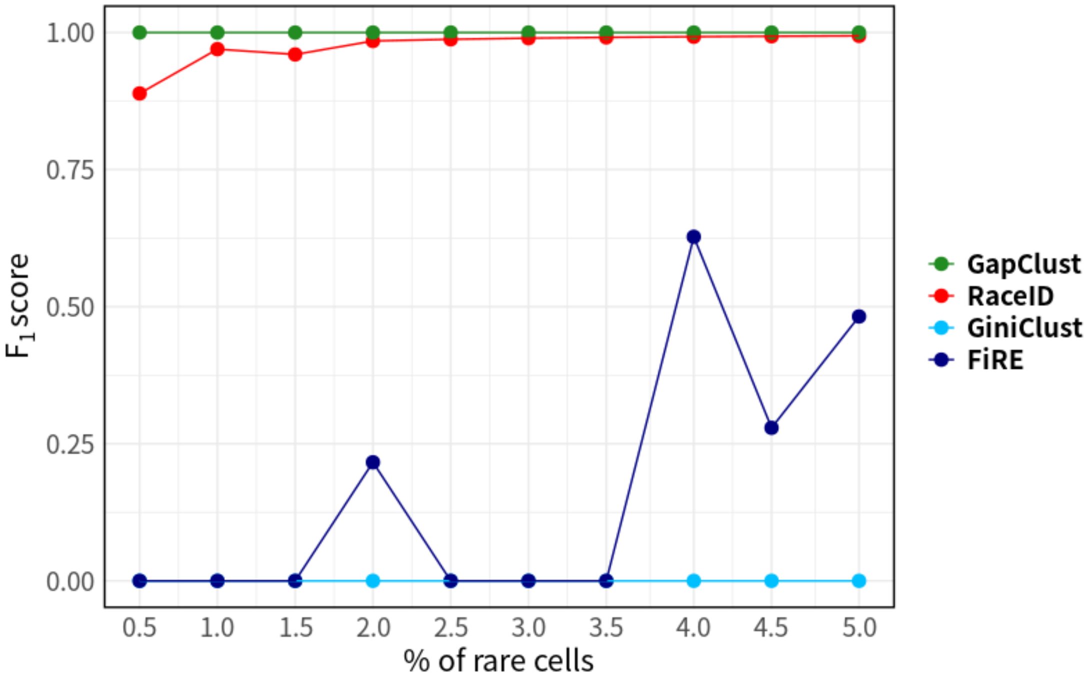

Supplementary Figure 7 Performance comparison of GapClust to RaceID, GiniClust and FiRE in detection of rare Jurkat cells in the ten simulated datasets subsampled from the 293T-Jurkat dataset with the rare cell type proportions ranging from 0.5% to 5.0%, quantified by F_1 score.

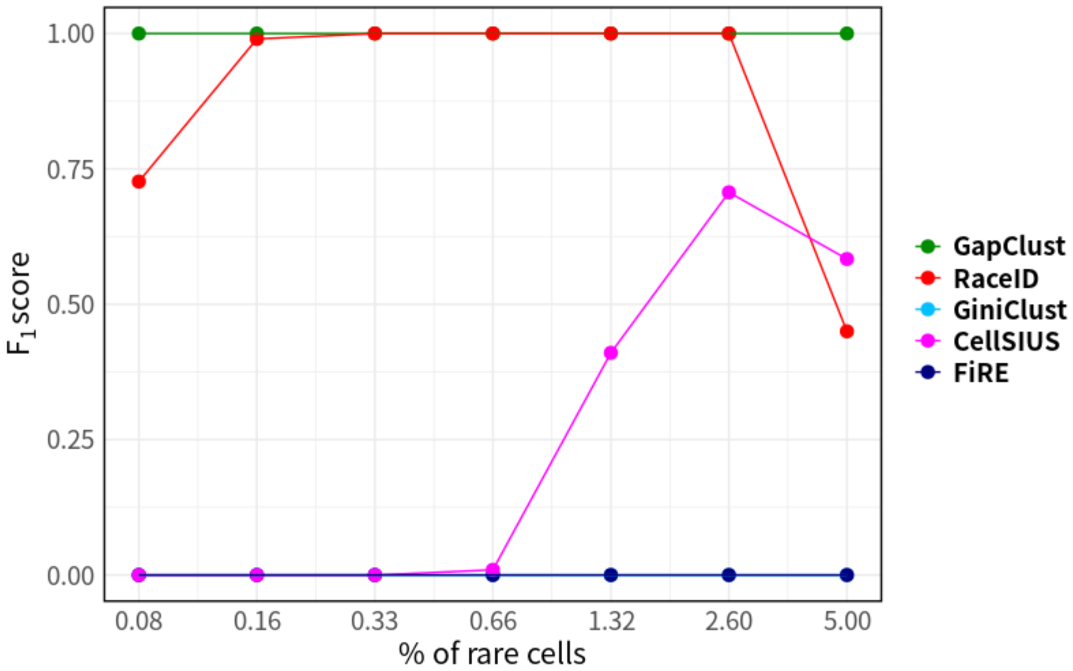

Supplementary Figure 8 Performance comparison of GapClust to RaceID, GiniClust, CellSIUS and FiRE in detection of monocyte doublets in 140 simulated datasets subsampled from the 68 k PBMC dataset with the rare cell type proportions ranging from 0.08% to 5.0%, quantified by F_1 score.

Supplementary Note 1: Comparison with EDGE in rare cell type identification

Firstly, we compare GapClust with EDGE[6] using simulated datasets with balanced and unbalanced major cell types. We generated the simulated data using Splatter[5] under four scenarios (Supplementary Table 1). Scenario 1: One thousand cells in three groups, with the ratio of 49.5:49.5:1 in group size (number of cells) and a low dropout rate in the expression of 5,000 genes; Scenario 2: Five thousand cells in three groups, with the ratio of 49.9:49.9:0.2 in group size (number of cells) and a low dropout rate in the expression of 5,000 genes; Scenario 3: One thousand cells in three groups, with the ratio of 66:33:1 in group size (number of cells) and a low dropout rate in the expression of 5,000 genes; Scenario 4: Five thousand cells in three groups, with the ratio of 66.4:33.4:0.2 in group size (number of cells) and a low dropout rate in the expression of 5,000 genes. The proportions of differentially expressed (DE) genes in Scenario 1 to 4 were fixed at 40%. Of note, in Scenario 1 and Scenario 2, the two dominant cell types are balanced in cell numbers, while in Scenario 3 and Scenario 4, the two dominant cell types are unbalanced in cell numbers (Supplementary Table 1).

Supplementary Table 1 Settings in simulation studies in comparison with EDGE.

	**Total No. of cells**	**Group proportions (%)**	**DE gene proportion (%)**
Scenario 1	1,000	(49.5, 49.5, 1)	40
Scenario 2	5,000	(49.9, 49.9, 0.2)	40
Scenario 3	1,000	(66, 33, 1)	40
Scenario 4	5,000	(66.4, 33.4, 0.2)	40

Of note, EDGE algorithm provides only the two-dimensional embeddings of the cells in the input single cell RNA sequencing (scRNA-seq) dataset, without reporting the group information of the rare cell types directly. User has to learn the rare cell types visually by the minor clusters in the two-dimensional embeddings, which can be biased. We use DBSCAN algorithm to identify the clusters in the two-dimensional embeddings learned by EDGE with tuned parameters (eps = 0.5, minPts = 5). Notably, DBSCAN can capture minor clusters through density-based spatial clustering with tuned parameters, while distance-based clustering algorithms such as *k*-means always neglect minor clusters.

The Supplementary Figure 9 displays the two-dimensional embeddings of simulated datasets learnt by EDGE in four scenarios, demonstrating that EDGE successfully separates all rare cells (the rare cell type, in red) from the abundant cells (the dominant type, in gray). We observe that rare cells identified by GapClust is perfectly consistent with the true rare cell type in all four scenarios (Supplementary Table 2). Meanwhile, EDGE can also identify the rare cell type perfectly in combination with DBSCAN method (Supplementary Table 2). The results show that both GapClust and EDGE are capable of accurate identification of rare cells in simulated datasets.

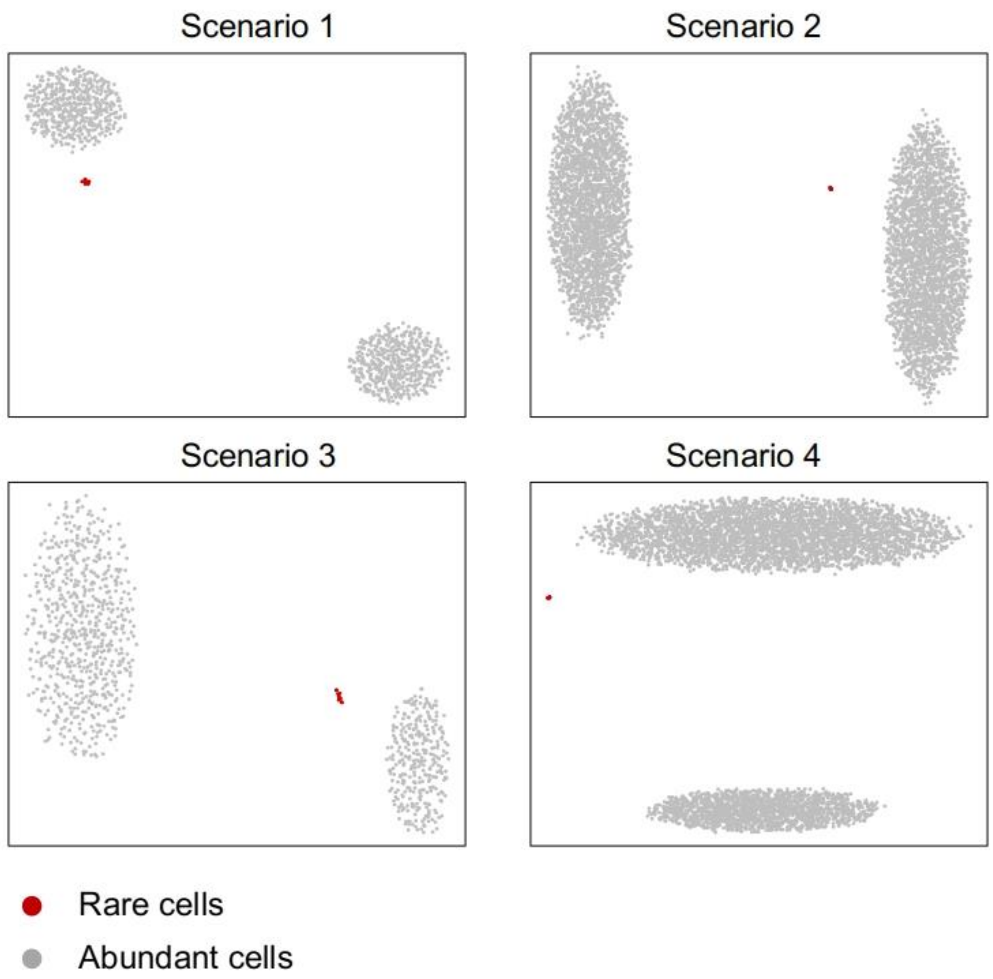

Supplementary Figure 9 The two-dimensional embeddings learnt by EDGE in Scenario 1, Scenario 2, Scenario 3, and Scenario 4. In each panel, the dots in red colour represent the rare cells, and the gray dots denote the abundant cell type.

Secondly, we compare GapClust with EDGE using simulated datasets with different differential expression levels between the rare cell type and the abundant cell type. Here, we generate eight simulation datasets consisting of 3 rare cells and 500 abundant cells by Splatter with varying de.prob parameters (the probability that a gene is differentially expressed in the rare cell group) including extremely low: 0.01, 0.02, 0.05, 0.1, moderate: 0.2, 0.4 and high: 0.6, 0.8 to mimic the different differential expression levels. Then EDGE and GapClust are both applied to the datasets. DBSCAN is used with the tuned parameters (eps = 0.5, minPts = 3). Here, the minPts is set as 3 rather than the default value 5, as the rare cell type contains only 3 cells.

Supplementary Table 2 The confusion matrices of the identified rare cell type with the true cell type in simulation studies of the four scenarios.

		Predicted cell type by **GapClust**			
Actual cell type		Rare cells	Abundant cells	Rare cells	Abundant cells
		**Scenario 1**		**Scenario 2**	
	Rare cells	12	0	11	0
	Abundant cells	0	988	0	4,989
		**Scenario 3**		**Scenario 4**	
	Rare cells	12	0	11	0
	Abundant cells	0	988	0	4,989
		Predicted cell type by **EDGE**			
		**Scenario 1**		**Scenario 2**	
	Rare cells	12	0	11	0
	Abundant cells	0	988	0	4,989
		**Scenario 3**		**Scenario 4**	
	Rare cells	12	0	11	0
	Abundant cells	0	988	0	4,989

In the two-dimensional embeddings, we find that EDGE clearly differentiates the three rare cells (red) from the remaining cells (gray) in the datasets with high differential expression levels (de.prob = 0.4, 0.6 and 0.8) (Supplementary Figure 10). In the scenarios with lower differential expression levels, the two-dimensional embeddings learnt by EDGE cannot distinguish the three rare cells from the remaining cells (Supplementary Figure 10). GapClust cannot identify the rare cell type when de.prob parameter is 0.01 and 0.02, but obtain improved performance with higher differential expression level (de.prob = 0.05) (Supplementary Figure 11). When de.prob is higher than 0.05, GapClust can detect the rare cell type perfectly (Supplementary Figure 11). In contrast, EDGE can detect the rare cell type perfectly only when de.prob is higher than 0.1, but fails when de.prob is 0.05 and 0.1(Supplementary Figure 11). Taken together, GapClust outperforms EDGE in scenarios with low differential expression level (de.prob = 0.05, 0.1) (Supplementary Figure 11), implying higher sensitivity of GapClust to differential expressed genes between the rare cell type and abundant cell type.

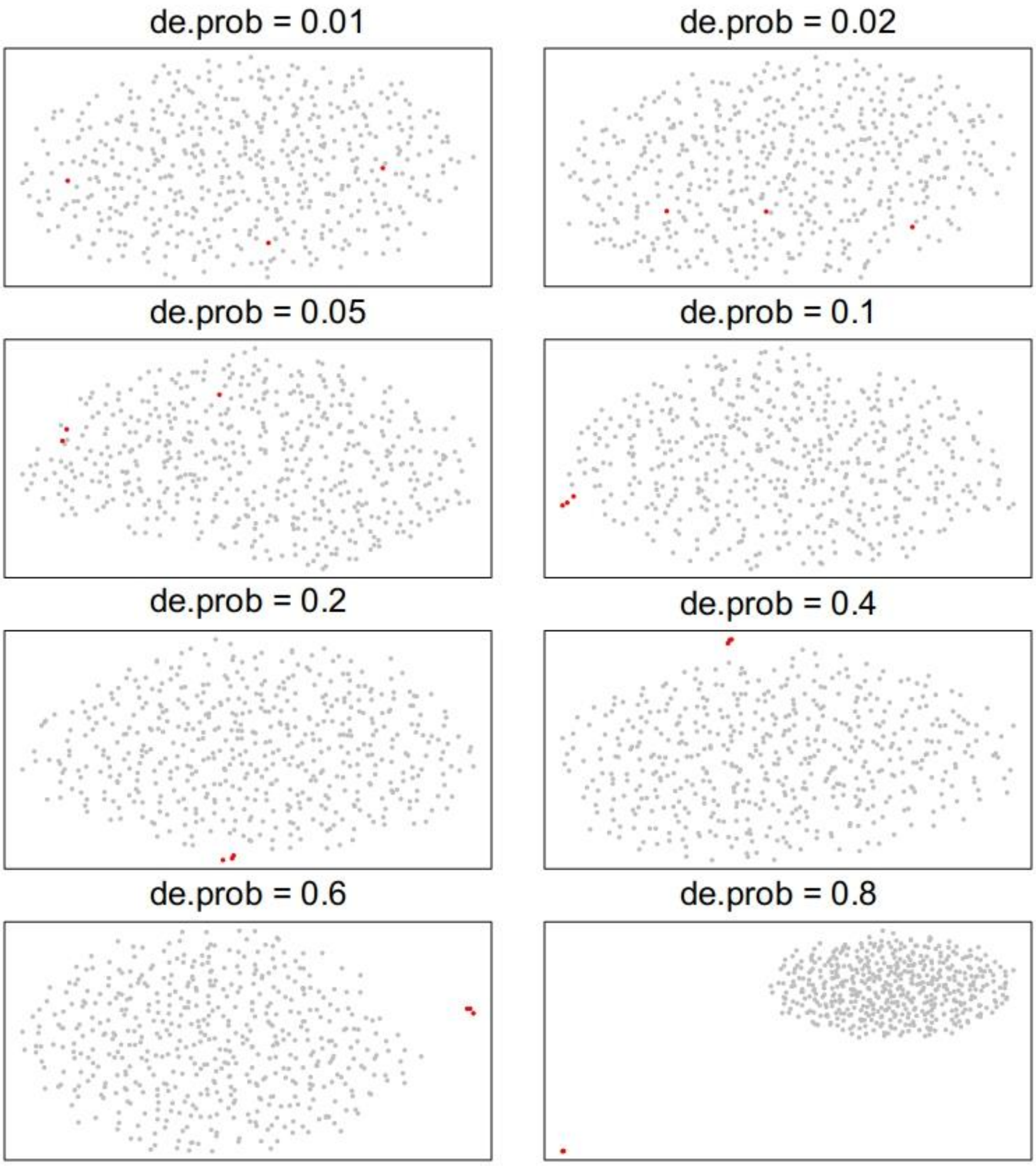

Supplementary Figure 10 The two-dimensional embeddings learnt by EDGE in the eight simulation datasets. The subplots represent different differential expression levels between the rare cell type and the abundant cell type. In each panel, the dots in red colour represent the rare cells, and the gray dots denote the abundant cell type.

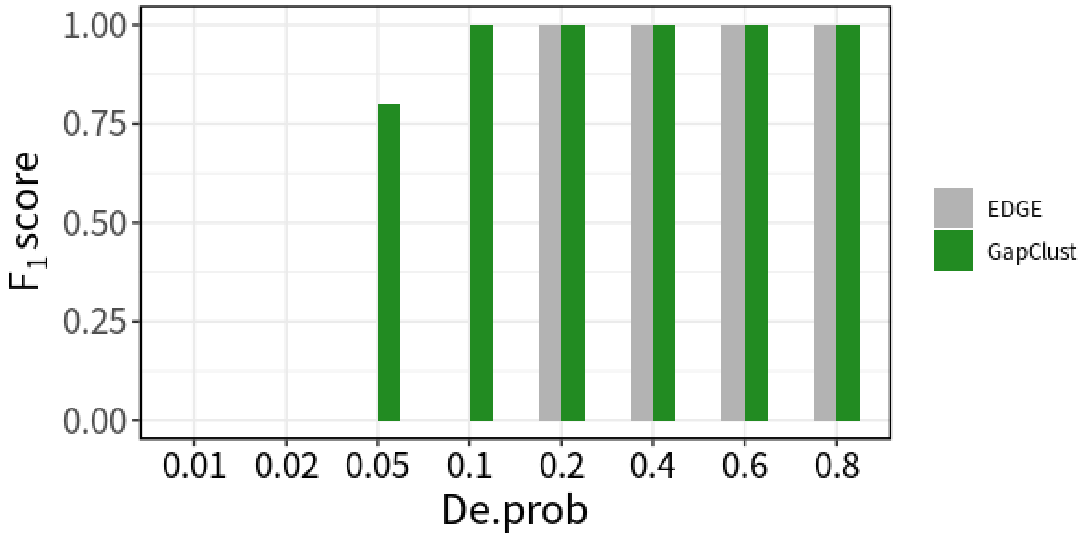

Supplementary Figure 11 The performance of GapClust and EDGE in the eight simulation datasets. The horizontal axis represents the differential expression level (de.prob parameter in Splatter) between the rare cell type and abundant cell type. The vertical axis represents the performance in the F_1 score metrics.

Thirdly, we compare GapClust with EDGE using simulated datasets with different rates of dropout events. Here, we generate nine simulation datasets consisting of 3 rare cells and 500 abundant cells by Splatter with different rates of dropout events, including 0.1, 0.2, 0.3, 0.4, 0.5, 0.6, 0.7, 0.8 and 0.9. Then EDGE and GapClust are both applied to the datasets. Similarly, DBSCAN is used with the tuned parameters (eps = 0.5, minPts = 3). Here, the minPts is set as 3 rather than the default value 5, as the rare cell type contains only 3 cells.

In the two-dimensional embeddings, we find that EDGE clearly differentiates the three rare cells (red) from the abundant cell type (gray) in the datasets with low rates of dropout events (0.1, 0.2, 0.3) (Supplementary Figure 12). In the scenarios with higher rates of dropout events, the two-dimensional embeddings learnt by EDGE cannot distinguish the three rare cells from the remaining cells (Supplementary Figure 12). GapClust cannot identify the rare cell type only when the rate of dropout events is extremely high (0.9), but detect the rare cell type perfectly in other datasets (Supplementary Figure 13). In contrast, EDGE can only detect the rare cell type perfectly in datasets with low and moderate rates of dropout events (0.1, 0.2, 0.3, 0.5) (Supplementary Figure 13). Taken together, GapClust performs better than EDGE in scenarios with high rates of dropout events (0.6, 0.7, 0.8).

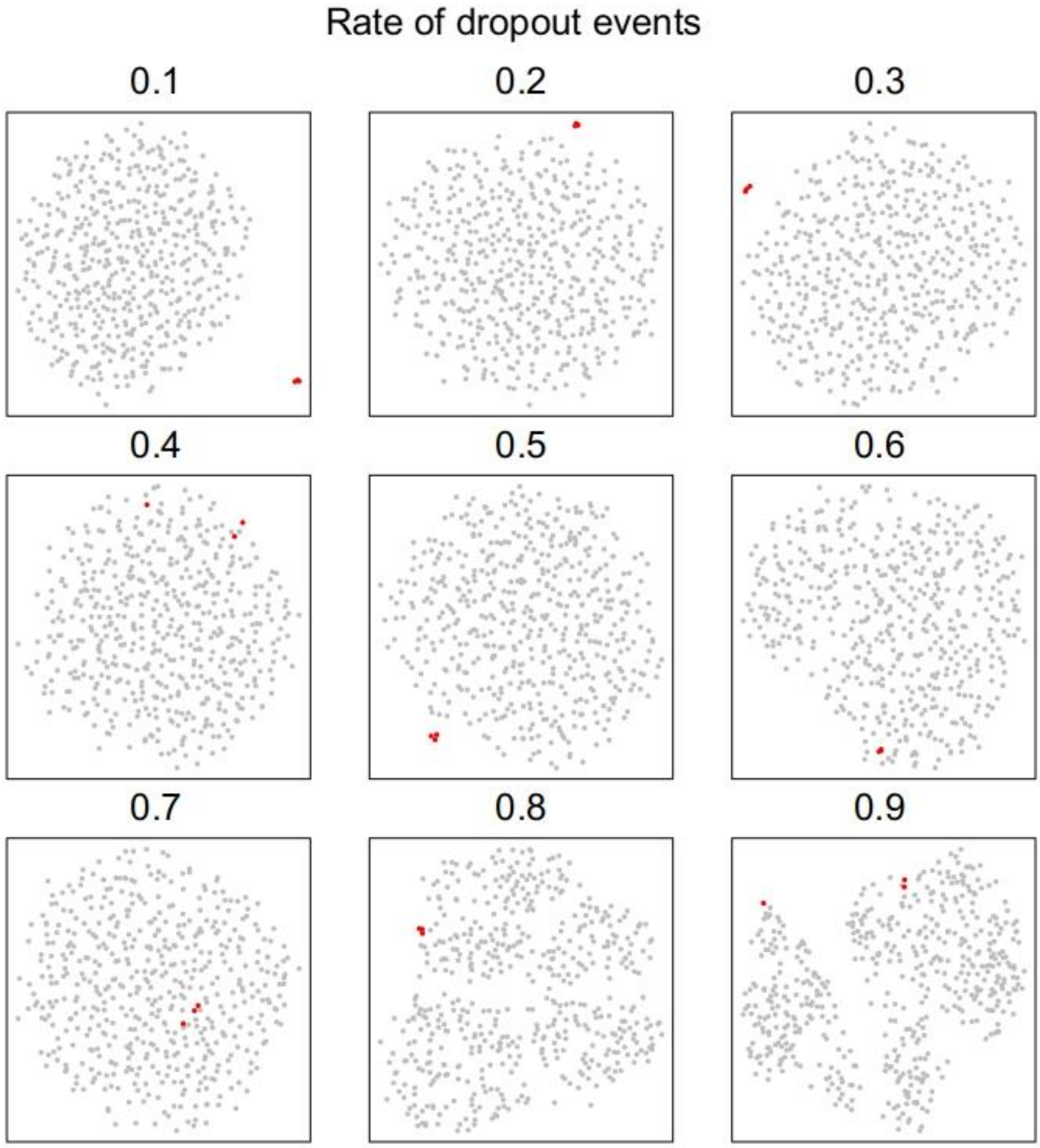

Supplementary Figure 12 The two-dimensional embeddings learnt by EDGE in the nine simulation datasets with different rates of dropout events by Splatter. The subplots represent different rates of dropout events ranging from 0.1 to 0.9. In each panel, the dots in red colour represent the rare cells, and the gray dots denote the abundant cell type.

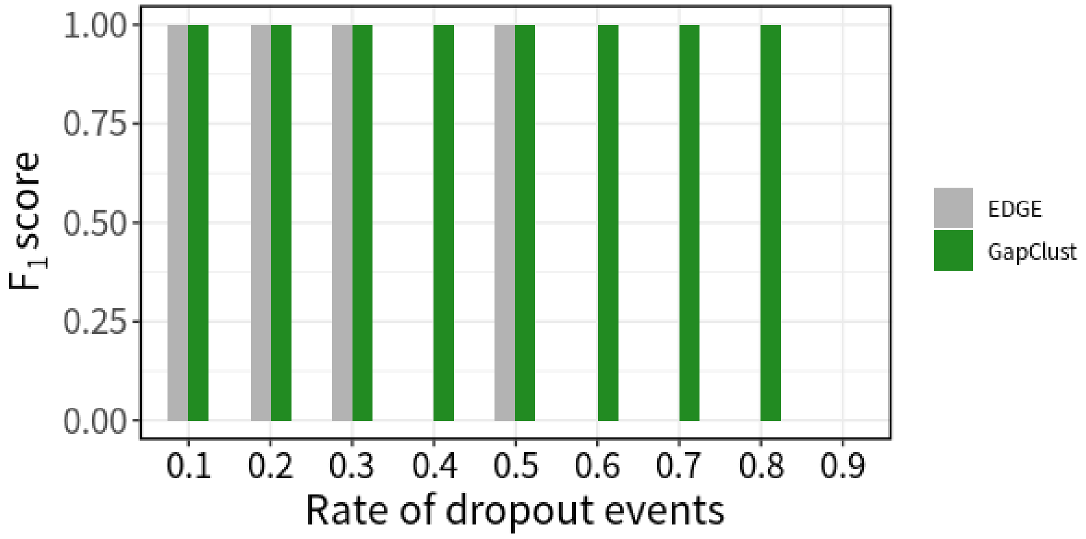

Supplementary Figure 13 The performance of GapClust and EDGE in the nine simulation datasets. The horizontal axis represents the different rates of dropout events ranging from 0.1 to 0.9. The vertical axis represents the performance in the F_1 score metrics.

Supplementary Table 3 GapClust is fast. Execution time (in minutes) recorded for the five methods while varying the number of cells from 1k to ~68k. RaceID is extremely slow for datasets with over 5 k cells, and thus applied to datasets with no larger than 35 k cells. GiniClust reported a runtime error, when the input expression profiles increased beyond ~45 k. CellSIUS failed when the input expression profiles increased beyond ~60 k.

	Computation time (min)				
Samples	**GapClust**	**RaceID**	**GiniClust**	**CellSIUS**	**FiRE**
1,000	0.014113	0.505467	0.185727	0.389149	0.020999
2,500	0.017316	3.928398	0.414181	0.72508	0.029166
5,000	0.035899	23.99051	0.709898	1.247367	0.054821
10,000	0.049924	78.31832	1.776898	2.444603	0.115114
15,000	0.073657	234.9635	3.494861	3.877048	0.180285
20,000	0.093468	560.6612	4.548236	5.401201	0.238741
25,000	0.108278	915.1861	5.96402	6.450866	0.321157
30,000	0.134334	1,131.709	7.388584	8.372198	0.393198
35,000	0.160736	2,782.132	8.969811	10.39575	0.492049
40,000	0.172991	-	11.27764	13.34094	0.538345
45,000	0.19102	-	15.03045	15.53882	0.639691
50,000	0.216493	-	-	17.99655	0.712395
55,000	0.238121	-	-	20.01057	0.758952
60,000	0.27343	-	-	24.83689	0.83293
65,000	0.283308	-	-	-	0.969287
68,579	0.307871	-	-	-	1.063907

Supplementary Table 4 GapClust is memory efficient. Maximum memory usage (in GB) recorded for the five methods while varying the number of cells from 1k to ~68k.

	**GapClust**	**RaceID**	**GiniClust**	**CellSIUS**	**FiRE**
Maximum samples processed	68,579	35,000	45,000	60,000	68,579
Maximum memory utilized (GB)	2.79	78.58	117.93	168.16	19.47

Supplementary Note 2: The human hippocampus dataset (GSE131258)

We apply GapClust to an existing scRNA-seq data containing ~30 k cells profiled from the human hippocampus (region of the brain that is associated primarily with memory) at gestational weeks 16-27[7]. Besides the doublets, GapClust identifies 390 rare cells yielding 3 cell subpopulations. We labeled these cell groups as R1-3 (Supplementary Figure 14a). Cell counts in these clusters are 94, 107 and 189, respectively.

The cell-type identity of the newly detected clusters was discovered by referring to the expression of known cell-type markers reported by Zhong et al[7]. (Supplementary Figure 14b). The markers *ASCL1*, *NEUROD2*, *GAD1*, *OLIG2*, *MBP*, *AQP4*, *SPARC* and *PTPRC* represent progenitor cells, excitatory neurons, inhibitory neurons, oligodendrocyte progenitor cells, oligodendrocytes, astrocytes, endothelial cells, and microglia, respectively[7]. Rare clusters R1 and R2 originate from the endothelial cells with *SPARC* gene highly expressed, and rare cluster R3 emerged from the cluster containing astrocytes with positive expression of the *AQP4* gene.

We perform differential expression analysis to find cell-type specific genes for the rare cell populations. Up-regulated genes are found in each cluster, clearly distinguishing it from the remaining cell types (Supplementary Figure 14c). Cells from the cluster R1 show high expression of *CLDN5*[8] and *MFSD2A*[9], which encode proteins involving the establishment of the blood-brain barrier, implying the crucial role of this cell population in the central nervous system. The rare cluster R2 emerges as a sub-type of endothelial cells by expressing growth factor encoding gene *IGF2* and *PTN*, a secreted growth factor that induces neurite outgrowth[10] and is mitogenic for fibroblasts, epithelial, and endothelial cells[11], demonstrating the growth-promoting capability of the cluster R2. Also, the high-level expressions of decorin encoding gene *DCN* and collagen type genes including *COL1A2* and *COL3A1* imply that these cells are closely associated with the setup of extracellular matrix (ECM)[12]. We find the rare cluster R3 with a wide spectrum of astrocyte markers including *CKB*[13], *PHDGH*[14] and *FOXJ1*[15].

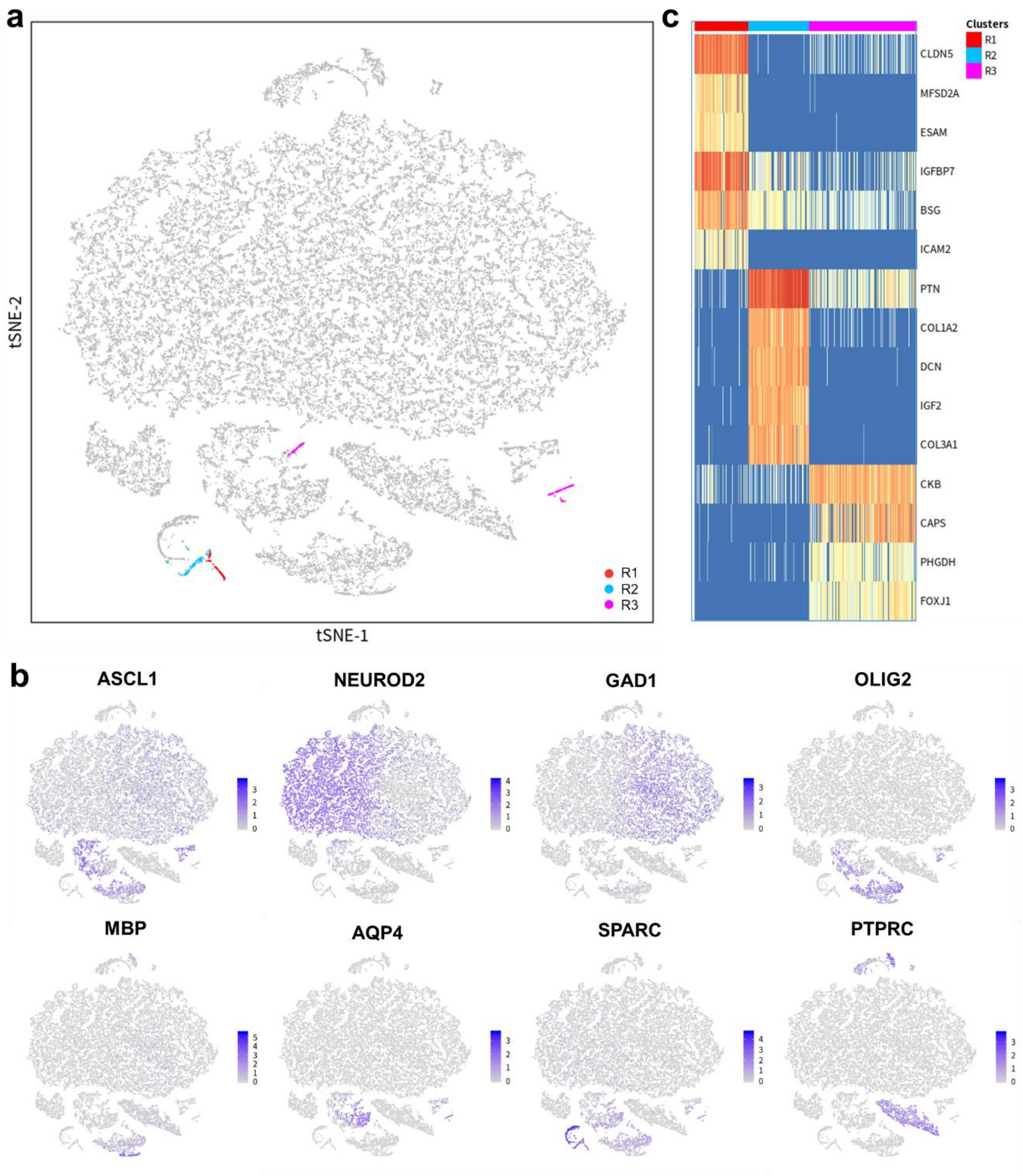

Supplementary Figure 14 Identification of rare cell types from the hippocampus dataset. **a** The tSNE plot of the full hippocampus dataset with the three rare cell clusters labeled in different colours. **b** The tSNE plots of the full hippocampus dataset displaying the expression distribution of eight cell-type specific markers across the whole cell population. **c** Heatmap of top differentially expressed genes between the three rare cell types.

Supplementary Note 3: The *Drosophila* wing disc dataset (GSE155543)

To evaluate the influence of batch effect, we have applied GapClust to a single-cell transcriptomics of a temporal cell atlas of the *Drosophila* wing disc from two developmental time points (96h and 120h)[16]. Two biological replicates were obtained at each time point that, generated data from 9,181 and 9,255 cells in the 96h samples, 10,401 and 7,160 cells in the 120h samples. The authors recovered two major cell types including adult muscle precursors and wing disc epithelial cells, and small numbers of tracheal cells and hemocytes in the combined dataset. Meanwhile, the authors reported the marker genes of hemocytes including *He* and *regucalcin*, and the marker genes of tracheal cells including *wat* and *tpr*[16]. Taken together, these datasets with replicates are ideal to benchmark GapClust on batch effect.

Besides the doublets identified, GapClust identifies 1, 1, 3 and 1 rare cell type in the four datasets, respectively. For the third dataset (120h), the rare cell types R2 and R3 express none of the marker genes reported by the authors, we thus focus on the rare cell type R1 of the four datasets. First, we use the marker genes mentioned above to determine the cell type of the rare cells in R1 identified in each dataset. High expressions of marker genes *He* and *regucalcin* in the cluster R1 of all datasets confirm that the cells of the cluster R1 are hemocytes across the datasets (Supplementary Figure 15a-c). However, the rare cell type of tracheal cells reported by the authors is not detected by GapClust in all datasets. To demonstrate whether small numbers of tracheal cells exist in these datasets, we explore the expression distribution of the reported marker genes including *wat* and *tpr*, and observe that these two genes are highly expressed in different patterns (Supplementary Figure 15d, e): the *wat* gene is highly expressed in only a few cells (Supplementary Figure 15d), whereas the expression of *tpr* can be observed in most of the cells (Supplementary Figure 15e). Therefore, the *wat* gene is more reliable for the tracheal cell type identification.

Intuitively, we can observe the transcription of *wat* gene only in very few cells in the first (96h) and fourth (120h replicate) datasets (Supplementary Figure 15d). As none of the rare cell types identified express tracheal cell marker genes *wat* and *tpr*, we have thus examined the doublets in each dataset identified by GapClust and found that marker gene *wat* is highly expressed in the doublets (R2) of the fourth datasets (120h replicate) (Supplementary Figure 16a, b). In addition, we have explored the first dataset (96h), and confirmed that only one cell expressing high level of *wat* gene (normalized count > 10) (Supplementary Figure 16c), making a singleton rather than a cluster. Compared with the hemocyte marker gene *He*, the expression level of *wat* gene is considerably lower (Supplementary Figure 16c), making it difficult to identify the rare cell type of tracheal cells.

Except for the rare cell type R1, GapClust identifies other two rare cell types R2 and R3 expressing none of the marker genes above in the third dataset (120h) (Supplementary Figure 16d), which are not reported by the authors. We apply differential expression analysis between the three rare cell types (Supplementary Figure 16e): high expression of *regucalcin* gene is observed in the rare cell type R1 of hemocytes, which has been validated (Supplementary Figure 15c); the transcription of

the enhancer of *yellow* genes *Dmel/yellow-e3* and *Dmel/yellow*-e2 in the rare cell type R2 implies the effect of these cells on *Drosophila* pigmentation, which is crucial in male mating success[17]; the cells in the rare cell type R3 display high expression of multiple functionally unknown genes including *Dmel/CG13044* and *Dmel/CG15353*.

Taken together, we have successfully benchmarked GapClust on the *Drosophila* wing disc datasets with replicates, and identified the reported rare hemocytes in all four datasets from different batches, demonstrating the reliability of GapClust in regard to batch effect.

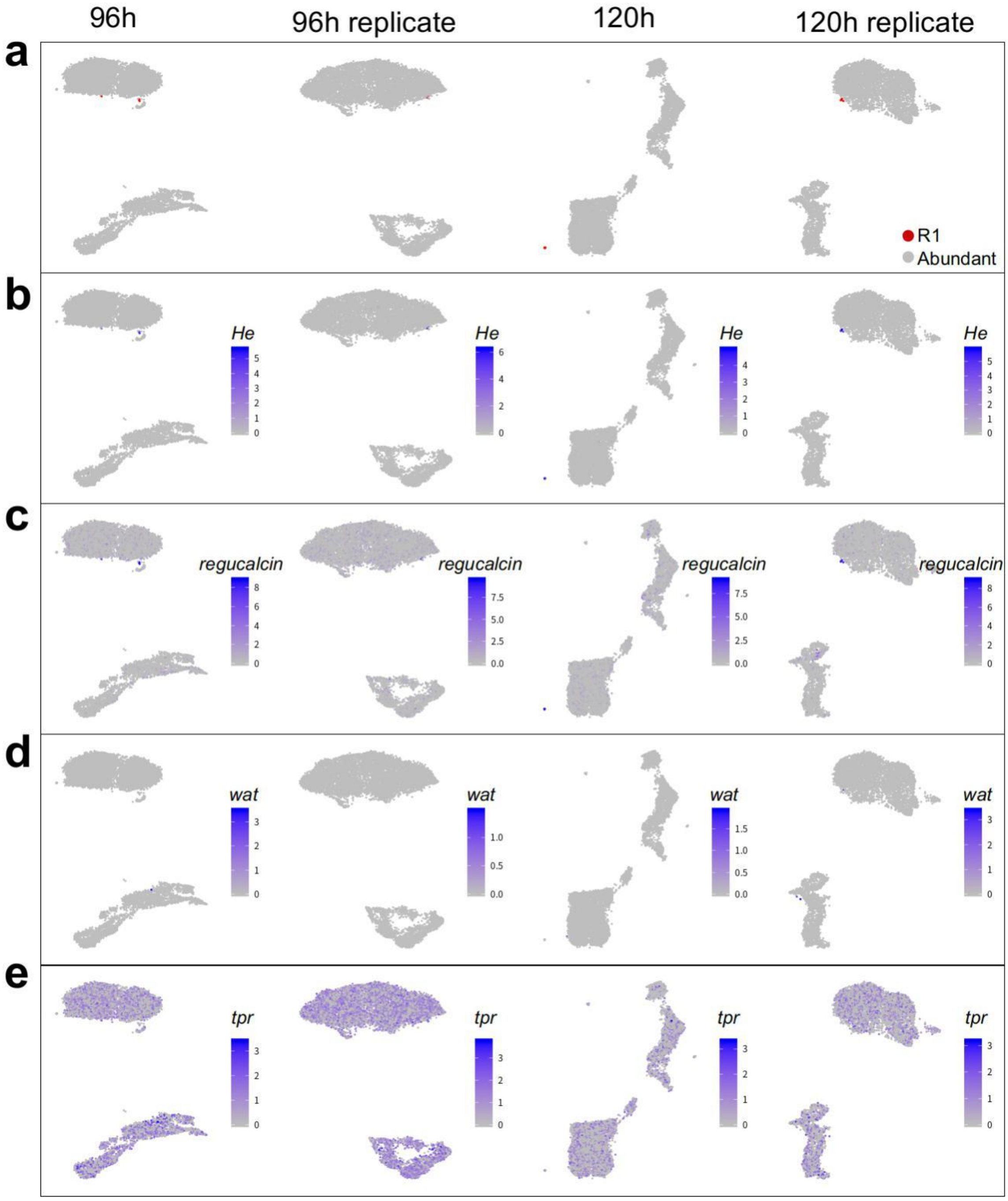

Supplementary Figure 15 Rare cell type identification with GapClust in *Drosophila* wing disc datasets. **a** The two-dimensional embeddings learnt by EDGE in the four *Drosophila* wing disc datasets. The dots in red colour represent the rare cell type (R1), while the dots in gray colour represent the abundant cell types. The two-dimensional embeddings learnt by EDGE of the four full datasets showing expression levels for the following genes: **b** hemocyte markers *Hemese* (*He*), **c** *regucalcin*, **d** tracheal cell markers *waterproof* (*wat*) and **e** *tracheal-prostasin* (*tpr*). Colour scales correspond to normalized (by total UMI) counts on a $\log_2$ scale.

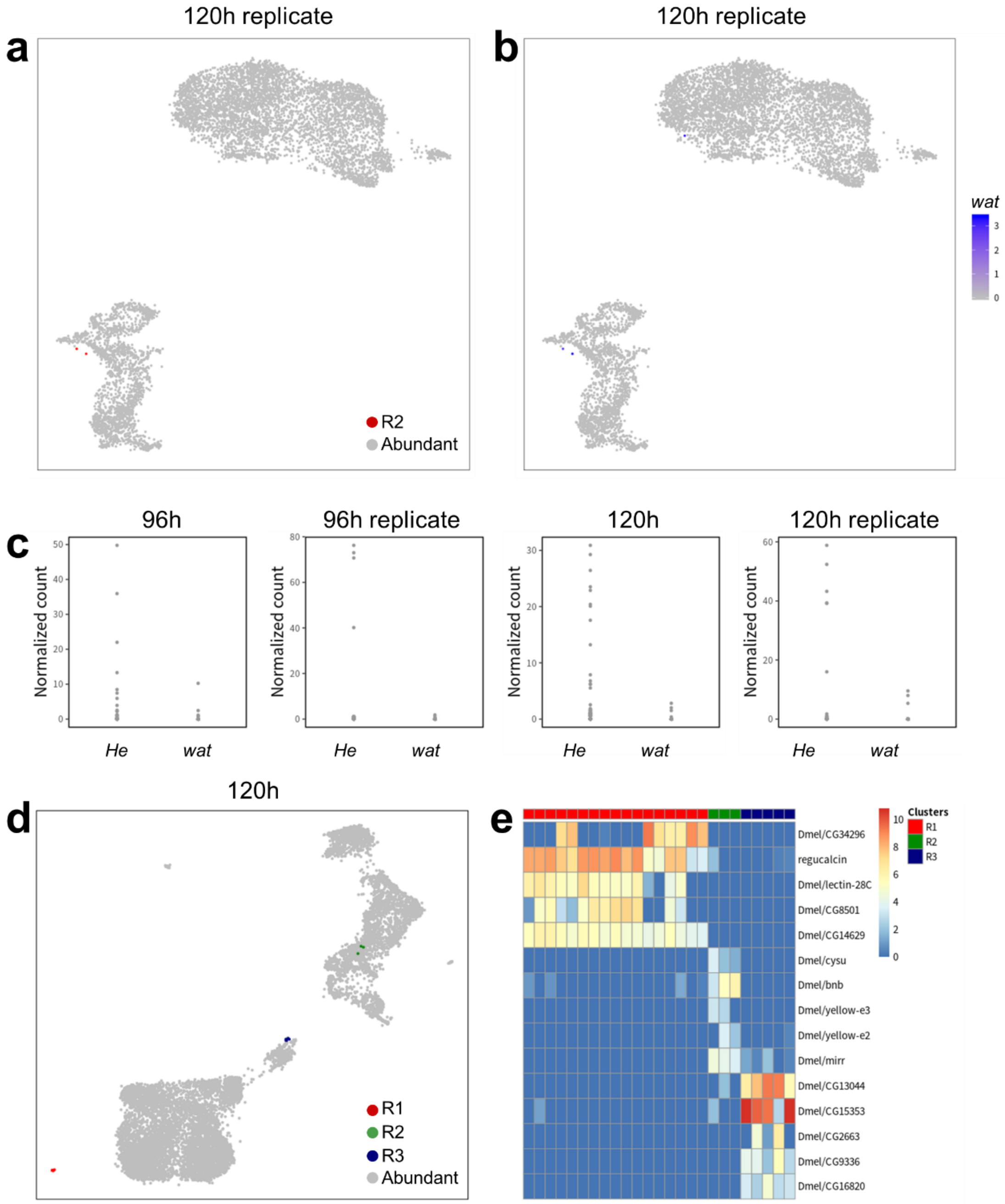

Supplementary Figure 16 Rare cell type identification with GapClust in *Drosophila* wing disc datasets. **a** The two-dimensional embeddings learnt by EDGE in the fourth *Drosophila* wing disc dataset (120h replicate). The dots in red represent the rare cell type (R2). **b** The two-dimensional embeddings learnt by EDGE of the fourth dataset (120h replicate) showing expression levels for the tracheal cell marker *waterproof* (*wat*). **c** The expression levels of marker genes *He* and *wat* in all four datasets. **d** The two-dimensional embeddings by EDGE in the third dataset (120h). The dots in red, green and blue colours represent the three rare cell types R1, R2 and R3. **e** The heatmap of top differentially expressed genes between the three rare cell types in the third dataset (120h).

Supplementary Note 4: The mouse ESC (embryonic stem cells) dataset (GSE65525)

The mouse embryonic stem cells (ESCs) were profiled at three time points: Day 0, Day 2, and Day 4 after leukemia inhibitory factor (LIF) removal induced differentiation[18]. We focused on a subset of 2,509 cells obtained from the Day 0 stage, where the cells remained undifferentiated. GapClust identified one rare cluster (R1) comprising 3 cells with high expression of genes from the *Zscan4* gene family (Supplementary Figure 17), which is consistent with the findings by the authors of GiniClust[2] and FiRE[4]. Expression of these genes has previously been observed in the two-cell embryo stage[19], although their potential function in pluripotency remains unknown.

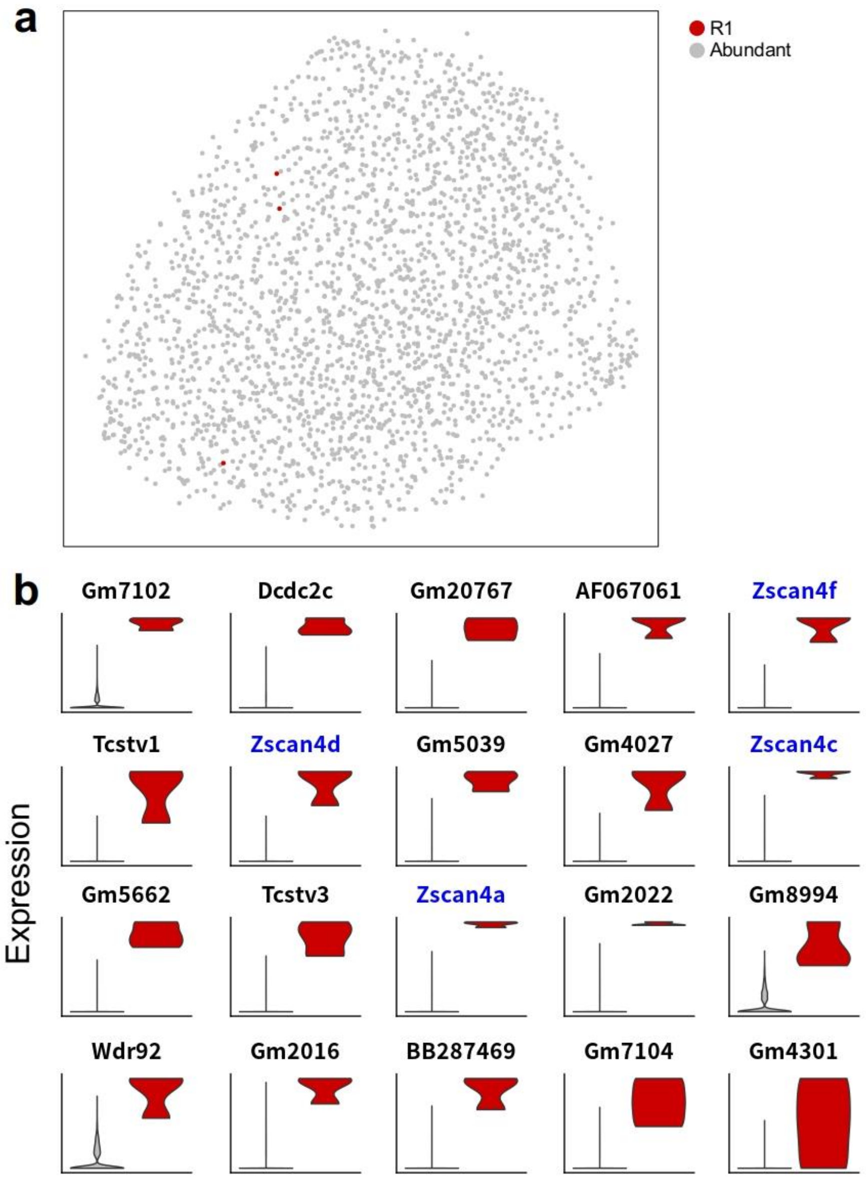

Supplementary Figure 17 Rare cell type identification in the mouse ESC (embryonic

stem cells) dataset. **a** The two-dimensional embeddings learnt by EDGE in the mouse ESC dataset. The dots in red colour represent the rare cell type (R1), and the dots in gray represent the abundant cell type. **b** Violin plots of top differentially expressed genes between the rare cell type R1 and abundant cell type. In each subplot, the gray group on the left represents the abundant cell type, and the red group on the right side represents the rare cell type R1. The genes in *Zscan4* gene family are labeled in blue colour. The same colour-coding scheme is used in all panels

Supplementary Note 5: The mouse somatosensory cortex dataset (GSE60361)

The 3,005 single cells were obtained from the mouse somatosensory cortex and hippocampus CA1 region[20]. GapClust identified six rare clusters (R1-6), comprising 3, 4, 6, 15, 26 and 38 cells, respectively (Supplementary Figure 18a). We have applied differential expression analysis between the rare cell types (Supplementary Figure 18b). Although the 3 (100%) cells in R1, 3 (50%) cells in R3 and 12 (80%) cells of R4 were annotated as astrocytes ependymal type by the authors (Supplementary Table 5), their expression profiles were distinct from each other. High expression of *Ttr*, *Igf2*, *Igfbp2* and *Enpp2* in R3 indicates the functional role in brain regions such as hippocampus[21-23]. We compare the 25 cells of R3 with the remaining oligodendrocytes and find that these cells display high expression of chemokine genes such as *Ccl2*, *Ccl3* and *Ccl7* (Supplementary Figure 18c), implying inflammatory roles in brain regions. Additionally, the 31 microglia cells of R6 are distinct from the remaining microglia cells in expression of genes such as *Ccl7*, *Pf4* and *Lyz2* (Supplementary Figure 18d).

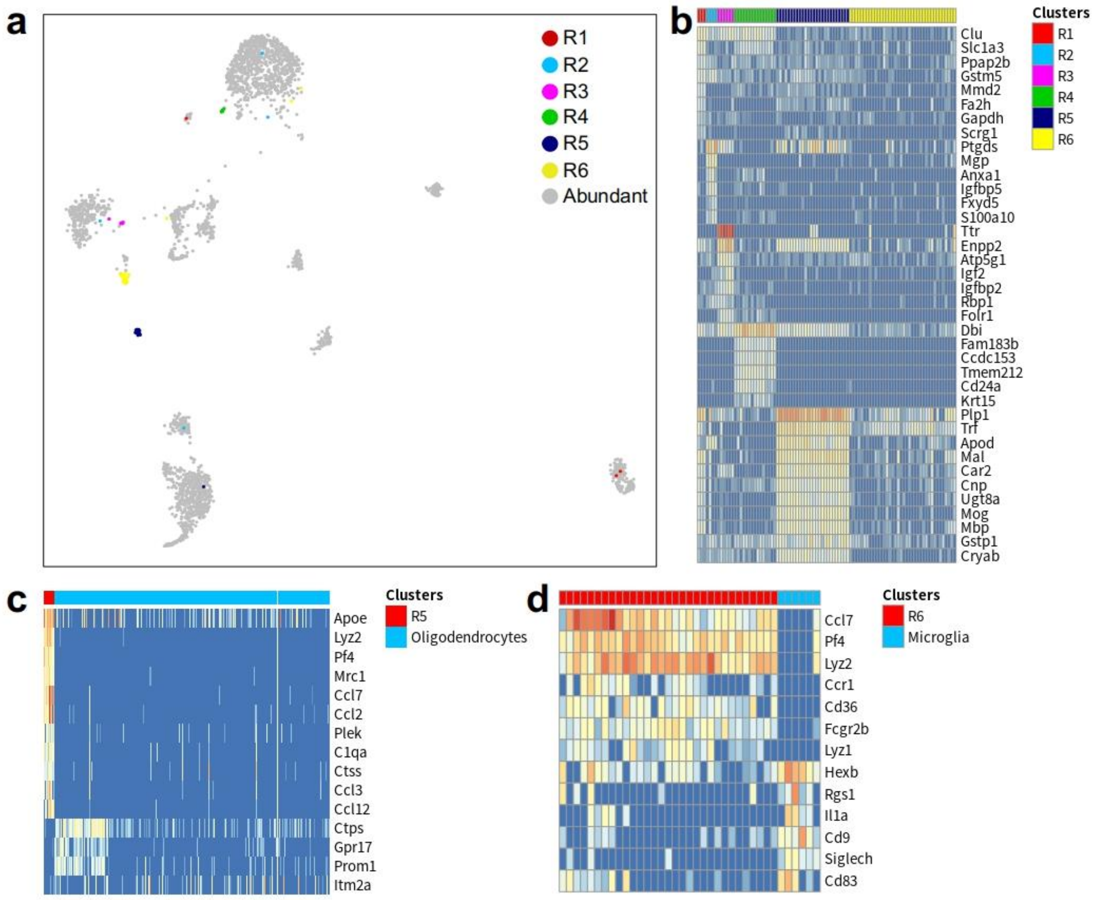

Supplementary Figure 18 GapClust identifies six rare cell types in the mouse somatosensory cortex dataset. **a** The two-dimensional embeddings learnt by EDGE in the mouse somatosensory cortex dataset. **b** The heatmap of top differentially expressed genes between the six rare cell types. **c** The heatmap of top differentially expressed genes between the rare cell type R5 and remaining oligodendrocytes in the

mouse somatosensory cortex dataset. **d** The heatmap of top differentially expressed genes between the rare cell type R6 and remaining microglia cells in the mouse somatosensory cortex dataset.

Supplementary Table 5 The cell types of the six rare cell clusters predefined by the authors[20].

	R1	R2	R3	R4	R5	R6
astrocytes_ependymal	3	0	3	12	0	0
endothelial-mural	0	2	0	1	0	0
interneurons	0	0	0	0	0	1
microglia	0	0	1	0	0	31
oligodendrocytes	0	1	1	0	25	0
pyramidal CA1	0	0	0	2	0	3
pyramidal SS	0	1	1	0	0	3

Supplementary Note 6: The Jurkat dataset

We evaluate GapClust using the dataset which comprises 293T and Jurkat cells at a rare cell (Jurkat cell) concentration of 2.5%[4]. GapClust identifies three rare clusters (R1, R2 and R3) in this dataset, which comprise 2, 13 and 45 cells, respectively (Supplementary Figure 19a-c). The expression profiles of the three rare types and the abundant cell type are distinct from each other (Supplementary Figure 19a, b). The R1 cluster includes only two cells, making itself a doublet. The rare cell type R2 comprising 13 cells was not identified by the authors of FiRE[4]. According to the rare cell concentration mentioned by the authors[4], the cells of R3 cluster belong to Jurkat cell type. The contributor mentioned that this data was mixed with only two cell types[4]. However, the three rare cell types detected by GapClust together with the abundant cell type suggest undiscovered heterogeneity within the dataset.

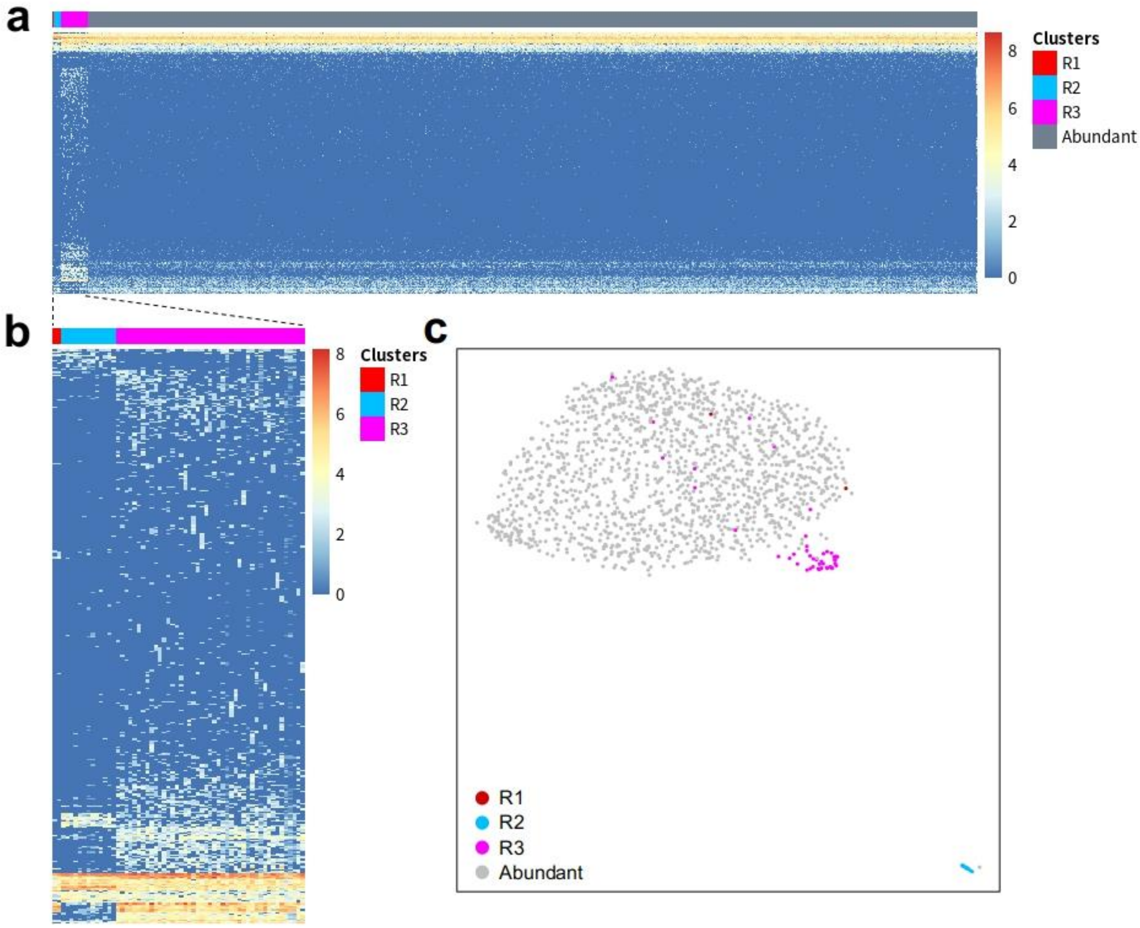

Supplementary Figure 19 Detection of rare cell types in subsampled 293T-Jurkat dataset of 1,580 cells. **a** The heatmap of top variable genes for the subsampled 293T-Jurkat dataset using the variance of standardized values. **b** The heatmap of top variable genes for the rare cell types in the subsampled 293T-Jurkat dataset using the variance of standardized values. **c** The two-dimensional embeddings learnt by EDGE in the subsampled 293T-Jurkat dataset. In each panel, the dots in red, blue, and magenta colours represent the three rare cell types (R1, R2 and R3), respectively.

Supplementary Note 7: The *Bacillus subtilis* cells dataset (GSE151940)

We apply GapClust to scRNA-seq data from the *Bacillus subtilis* cells sampled at different growth stages, creating a detailed atlas of changes in metabolism and lifestyle[24]. A total of four minor clusters comprising 2, 3, 4 and 189 cells are identified, and the first two rare cell types are similar to each other. We have thus combined these two minor clusters of cells into one single cluster of 5 cells for convenience. Here we denote the three clusters as R1, R2 and R3 (Supplementary Figure 20a). The OD (optical density) values are used to represent the growth curve. The five cells of R1 were sampled at OD2.0 to 2.7, and the four cells of R2 were profiled at early stages (OD0.4 to 1.0). The majority of the 189 cells in R3 were sampled at OD1.7.

We apply differential expression analysis among the three rare cell types (Supplementary Figure 20b). Transcription of genes in the inositol metabolism (*iolA*-*G*, *J*) in cluster R1 indicates the heterogeneous fashion in a subpopulation of cells between logarithmic and early stationary phases[24]. Most of the genes specifically expressed in cluster R2 represent known PBSX prophage genes with functions in phage replication (*xtmA* and *xtmB*), as well as PBSX associated genes of unknown function. Thus, we identified a rare subpopulation of cells in the state of prophage induction, which is consistent with the findings in the original research[24]. High expression of genes in the oligo-β-glucoside metabolism (*licA*, *licC* and *licH*) distinguishes the cells in the cluster R3 from the remaining cells, implying the cellular heterogeneity in logarithmic phase.

Furthermore, we investigate the change in cellular composition after filtering cells expressing at least 100 genes. For the collected OD points 0.4, 1.0, 1.7, 2.0, 2.7 and 3.2, the total numbers of cells sampled at each OD point are 5,202, 5,180, 4,699, 4,241, 3,437 and 2,698, while the numbers of the cells passing the criteria are 2,275, 1,920, 199, 37, 19 and 8, respectively, implying that the cells grown at the late stage of growth curve tend to express less genes.

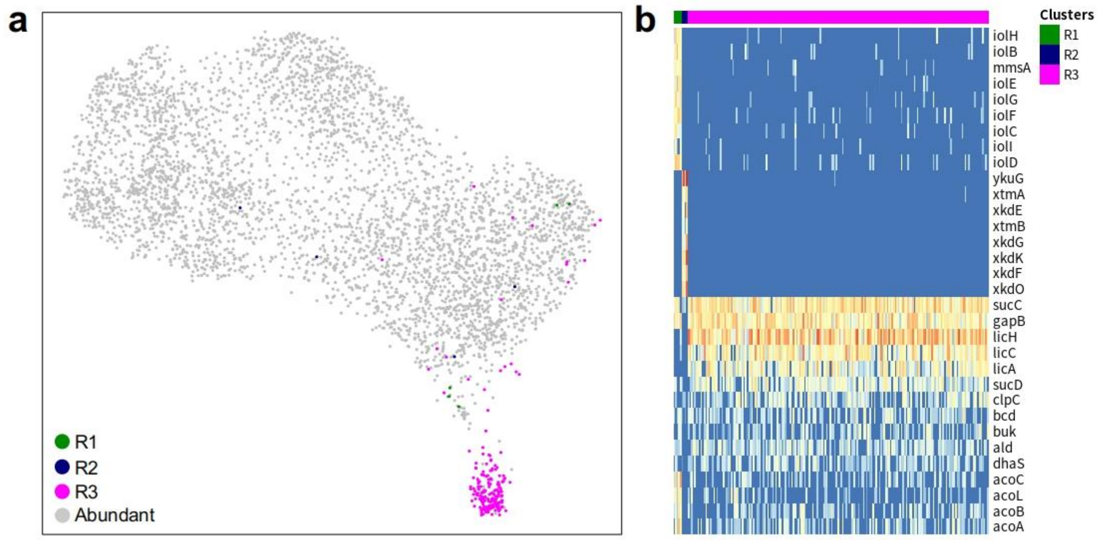

Supplementary Figure 20 GapClust identifies three rare cell types in the *Bacillus subtilis* cells dataset. **a** The two-dimensional embeddings learnt by EDGE in the *Bacillus subtilis* cells dataset. **b** The heatmap of top differentially expressed genes between the three rare cell types.

Supplementary Note 8: The mouse tracheal epithelial cells dataset (GSE103354)

We explore the heterogeneity of mouse tracheal epithelial cells by analyzing the scRNA-seq dataset (GSE103354)[25] comprising 7,194 cells using GapClust. In total, we have identified six rare cell clusters (R1-6) comprising 3, 7, 13, 22, 54 and 113 cells, respectively (Supplementary Figure 21a). The cluster R1 and R6 were annotated as tuft cells by the authors, and consolidated by the transcription of marker gene *Trpm5*[26] (Supplementary Figure 21b). However, differential expression of another tuft cell marker gene *Dclk1* as well as other genes including *Atp1a2*, *Vav1* and *Nkd1* demonstrates that these two rare cell types represent distinct tuft cell populations. The cells in cluster R2 were labeled as ciliated cells, which distinguished them from the remaining ciliated cells by high expression of cell cycle related genes such as *Cdk1*[27], *Smc4*[28], *Cdc20*[27], *Cks1b*[29] and *H2afx*[30] (Supplementary Figure 21c). Although the cells in the two clusters R3 and R4 were annotated as goblet cell type by the authors, the differential expression of *Dcpp3*, *Sbp1*, *Tff2*, *BC048546* and *Muc5* genes implied different sub-types. The cells in cluster R5 were grouped into pulmonary neuroendocrine cells. These rare cell types were reported by the authors[25], implying the reliability of GapClust in real dataset application.

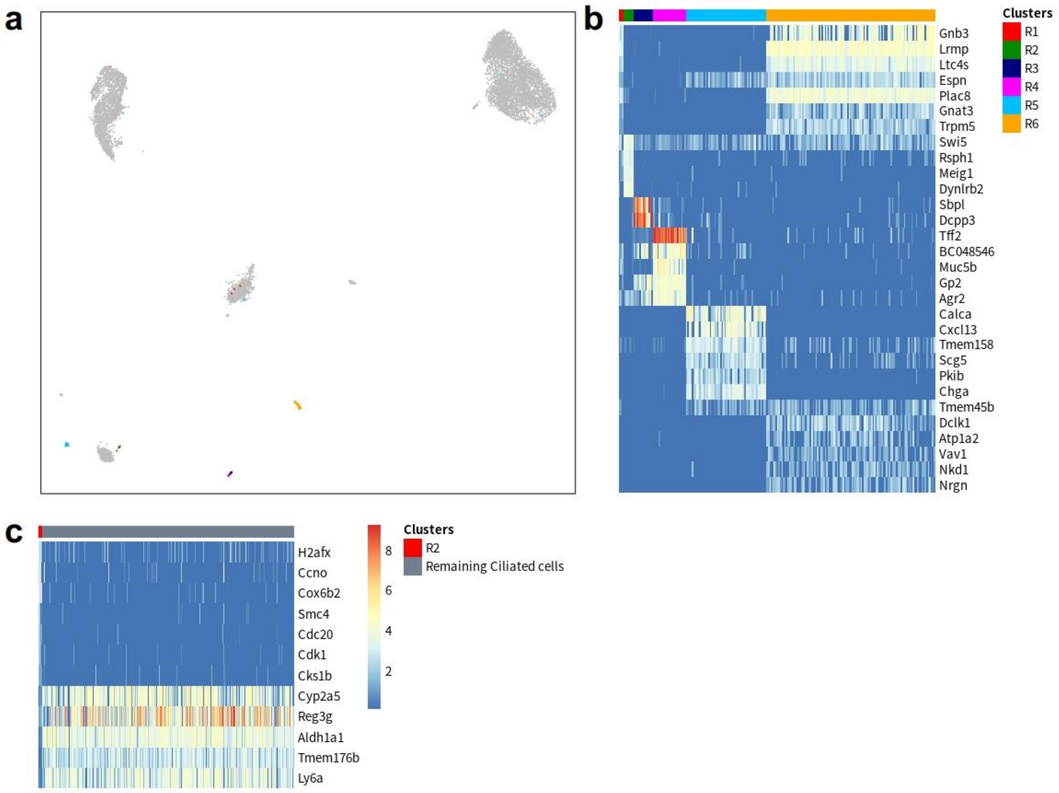

Supplementary Figure 21 GapClust identifies six rare cell types in the mouse tracheal epithelial cells dataset. **a** The two-dimensional embeddings learnt by EDGE in the mouse tracheal epithelial cells dataset. **b** The heatmap of top differentially expressed genes between the six rare cell types. **c** The heatmap of top differentially expressed genes between the rare cell type R2 and remaining ciliated cells in the mouse tracheal epithelial cells dataset.

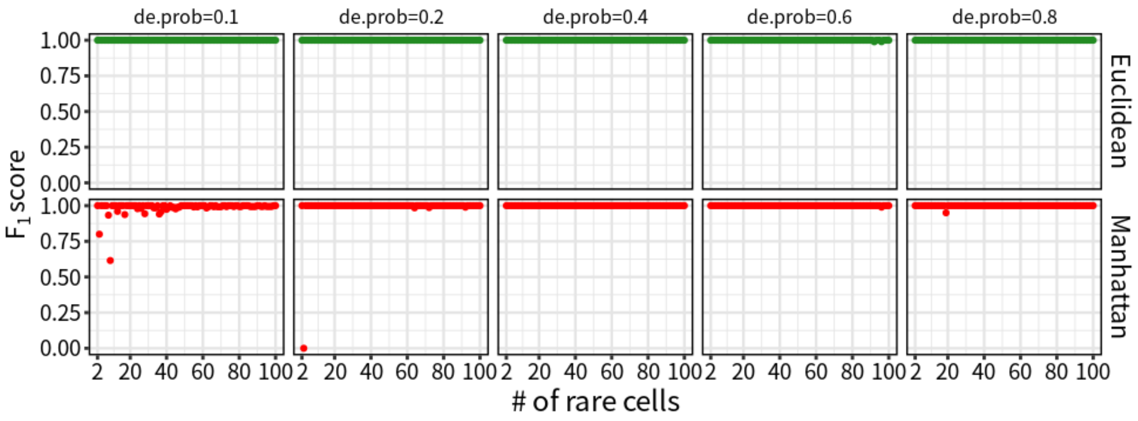

Supplementary Figure 22 The performance of GapClust using Euclidean and Manhattan distance. The five columns represent the results from the datasets with different levels of differential expression between the rare cell type and the abundant cell type by varying the de.prob parameter (0.1, 0.2, 0.4, 0.6 and 0.8) of Splatter.

Supplementary Reference

1 Grun, D. *et al.* De Novo Prediction of Stem Cell Identity using Single-Cell Transcriptome Data. *Cell Stem Cell* **19**, 266-277 (2016).

2 Tsoucas, D. & Yuan, G. GiniClust2: a cluster-aware, weighted ensemble clustering method for cell-type detection. *Genome Biology* **19**, 58-58 (2018).

3 Wegmann, R. *et al.* CellSIUS provides sensitive and specific detection of rare cell populations from complex single-cell RNA-seq data. *Genome Biology* **20**, 142-142 (2019).

4 Jindal, A., Gupta, P., Jayadeva & Sengupta, D. Discovery of rare cells from voluminous single cell expression data. *Nature Communications* **9**, 4719 (2018).

5 Zappia, L., Phipson, B. & Oshlack, A. Splatter: simulation of single-cell RNA sequencing data. *Genome Biology* **18**, 174 (2017).

6 Sun, X., Liu, Y. & An, L. Ensemble dimensionality reduction and feature gene extraction for single-cell RNA-seq data. *Nat Commun* **11**, 5853, doi:10.1038/s41467-020-19465-7 (2020).

7 Zhong, S. *et al.* Decoding the development of the human hippocampus. *Nature* **577**, 531-536, doi:10.1038/s41586-019-1917-5 (2020).

8 Haruwaka, K. *et al.* Dual microglia effects on blood brain barrier permeability induced by systemic inflammation. *Nat Commun* **10**, 5816, doi:10.1038/s41467-019-13812-z (2019).

9 Ben-Zvi, A. *et al.* Mfsd2a is critical for the formation and function of the blood–brain barrier. *Nature* **509**, 507 (2014).

10 Li, Y. S. *et al.* Cloning and expression of a developmentally regulated protein that induces mitogenic and neurite outgrowth activity. *Science* **250**.

11 Fang, W. J., Hartmann, N., Chow, D. T., Riegel, A. T. & Wellstein, A. Pleiotrophin stimulates fibroblasts and endothelial and epithelial cells and is expressed in human cancer. *Journal of Biological Chemistry* **267**, 25889-25897 (1992).

12 Long, K., Moss, L., Laursen, L., Boulter, L. & Ffrench-Constant, C. Integrin signalling regulates the expansion of neuroepithelial progenitors and neurogenesis via Wnt7a and Decorin. *Nature Communications* **7**, 10354 (2016).

13 Molloy, G. R., Wilson, C. D., Benfield, P., Devellis, J. & Kumar, S. Rat Brain Creatine Kinase Messenger RNA Levels Are High in Primary Cultures of Brain Astrocytes and Oligodendrocytes and Low in Neurons. *Journal of Neurochemistry* **59**, 1925-1932 (1992).

14 Liu, J., Guo, S., Li, Q., Yang, L. & Xia…, Z. Phosphoglycerate dehydrogenase induces glioma cells proliferation and invasion by stabilizing forkhead box M1. *Journal of Neuro-Oncology* **111**, 245-255 (2013).

15 Jacquet, B. V. *et al.* FoxJ1-dependent gene expression is required for differentiation of radial glia into ependymal cells and a subset of astrocytes in the postnatal brain. *Development* **136**, 4021-4031 (2009).

16 Everetts, N. J., Worley, M. I., Yasutomi, R., Yosef, N. & Hariharan, I. K. Single-cell transcriptomics of the Drosophila wing disc reveals instructive epithelium-to-myoblast interactions. *Elife* **10**, doi:10.7554/eLife.61276 (2021).
17 Massey, J. H., Chung, D., Siwanowicz, I., Stern, D. L. & Wittkopp, P. J. The yellow gene influences Drosophila male mating success through sex comb melanization. (2019).
18 Ayyaz, A. *et al.* Single-cell transcriptomes of the regenerating intestine reveal a revival stem cell. *Nature* **569**, 1-5 (2019).
19 Klein, A. M. *et al.* Droplet barcoding for single-cell transcriptomics applied to embryonic stem cells. *Cell* **161**, 1187-1201 (2015).
20 Zeisel, A. *et al.* Brain structure. Cell types in the mouse cortex and hippocampus revealed by single-cell RNA-seq. *Science* **347**, 1138-1142, doi:10.1126/science.aaa1934 (2015).
21 Arneson, D. *et al.* Single cell molecular alterations reveal target cells and pathways of concussive brain injury. *Nat Commun* **9**, 3894, doi:10.1038/s41467-018-06222-0 (2018).
22 Takeo, C., Ikeda, K., Horie-Inoue, K. & Inoue, S. Identification of Igf2, Igfbp2 and Enpp2 as estrogen-responsive genes in rat hippocampus. *Endocrinologia Japonica* **56**, 113-120 (2009).
23 Zhu, X. *et al.* RBM3 promotes neurogenesis in a niche-dependent manner via IMP2-IGF2 signaling pathway after hypoxic-ischemic brain injury. *Nat Commun* **10**, 3983, doi:10.1038/s41467-019-11870-x (2019).
24 Kuchina, A. *et al.* Microbial single-cell RNA sequencing by split-pool barcoding. *Science* **371**, doi:10.1126/science.aba5257 (2021).
25 Montoro, D. T. *et al.* A revised airway epithelial hierarchy includes CFTR-expressing ionocytes. *Nature* **560**, 319-324, doi:10.1038/s41586-018-0393-7 (2018).
26 Howitt, M. R. *et al.* Tuft cells, taste-chemosensory cells, orchestrate parasite type 2 immunity in the gut. *Science* **351**, 1329-1333 (2016).
27 Yang, Q. & Jr, J. The Cdk1–APC/C cell cycle oscillator circuit functions as a time-delayed, ultrasensitive switch. *Nature Cell Biology*.
28 Thadani, R., Kamenz, J., Heeger, S., Munoz, S. & Uhlmann, F. Cell-Cycle Regulation of Dynamic Chromosome Association of the Condensin Complex. *Cell Rep* **23**, 2308-2317, doi:10.1016/j.celrep.2018.04.082 (2018).
29 Zhan, F. *et al.* CKS1B, overexpressed in aggressive disease, regulates multiple myeloma growth and survival through SKP2- and p27Kip1-dependent and -independent mechanisms. *Blood* **109**, 4995-5001 (2007).
30 Cloutier, J. M. *et al.* Histone H2AFX Links Meiotic Chromosome Asynapsis to Prophase I Oocyte Loss in Mammals. *PLoS Genet* **11**, e1005462, doi:10.1371/journal.pgen.1005462 (2015).
