# Supplementary material for: GapClust is a light-weight approach distinguishing rare cells from voluminous single cell expression profiles
Source: Nat Commun. 2021 Jul 7;12:4197. doi: 10.1038/s41467-021-24489-8 (PMC8263561; doi:10.1038/s41467-021-24489-8)
Supplement: Supplementary file 2 — Reporting Summary [file 41467_2021_24489_MOESM2_ESM.pdf]

nature research

Corresponding author(s): Zhangsheng Yu

Last updated by author(s): May 17, 2021

Reporting Summary

Nature Research wishes to improve the reproducibility of the work that we publish. This form provides structure for consistency and transparency in reporting. For further information on Nature Research policies, see our Editorial Policies and the Editorial Policy Checklist.

Statistics

For all statistical analyses, confirm that the following items are present in the figure legend, table legend, main text, or Methods section.

n/a	Confirmed	
☐	☒	The exact sample size (*n*) for each experimental group/condition, given as a discrete number and unit of measurement
☒	☐	A statement on whether measurements were taken from distinct samples or whether the same sample was measured repeatedly
☐	☒	The statistical test(s) used AND whether they are one- or two-sided<br>*Only common tests should be described solely by name; describe more complex techniques in the Methods section.*
☒	☐	A description of all covariates tested
☒	☐	A description of any assumptions or corrections, such as tests of normality and adjustment for multiple comparisons
☒	☐	A full description of the statistical parameters including central tendency (e.g. means) or other basic estimates (e.g. regression coefficient) AND variation (e.g. standard deviation) or associated estimates of uncertainty (e.g. confidence intervals)
☒	☐	For null hypothesis testing, the test statistic (e.g. F, t, r) with confidence intervals, effect sizes, degrees of freedom and P value noted<br>*Give P values as exact values whenever suitable.*
☒	☐	For Bayesian analysis, information on the choice of priors and Markov chain Monte Carlo settings
☒	☐	For hierarchical and complex designs, identification of the appropriate level for tests and full reporting of outcomes
☒	☐	Estimates of effect sizes (e.g. Cohen's *d*, Pearson's *r*), indicating how they were calculated

Our web collection on statistics for biologists contains articles on many of the points above.

Software and code

Policy information about availability of computer code

Data collection	No software was used for data collection.
Data analysis	GapClust is implemented in R and the source code has been deposited at https://github.com/fabotao/GapClust and Zenodo (DOI: https://doi.org/10.5281/zenodo.4765514).<br>GiniClust2 package was obtained from GitHub (dtsoucas/GiniClust2, version as of 14 Jul 2018). The analysis was run with default parameters: MinPts = 3, eps = 0.45, k = 10 for all datasets, except that MinPts was adjusted to 2 in case of doublets identification, whilst other parameters were set to their defaults.<br>RaceID package was directly applied to the normalized expression matrix, with all parameters at their default values, except that the initial clusters were determined according to abundant cell types rather than by k-medoids.<br>CellSIUS package was downloaded from GitHub (Novartis/CellSIUS, version as of 3 Jun 2019). The initial major cell types were determined using k-means with a data-specific k. Other parameters were set to their defaults, except for the min_n_cells parameter, which was set as 2 in the case of detecting any doublets.<br>FiRE package was obtained from GitHub (princethewinner/FiRE, version as of 9 Aug 2019). All parameters were set to their defaults. As to IQR thresholding criteria for rare cell detection, we also tried 1.0 and 0.5 for the IQR coefficient.<br>EDGE package was obtained from GitHub (shawnstat/EDGE, version 1.0). All parameters were set to their defaults.

For manuscripts utilizing custom algorithms or software that are central to the research but not yet described in published literature, software must be made available to editors and reviewers. We strongly encourage code deposition in a community repository (e.g. GitHub). See the Nature Research guidelines for submitting code & software for further information.

Data

Policy information about availability of data

All manuscripts must include a data availability statement. This statement should provide the following information, where applicable:

- Accession codes, unique identifiers, or web links for publicly available datasets
- A list of figures that have associated raw data
- A description of any restrictions on data availability

The study uses multiple publicly available scRNA-seq datasets. Both 68 k PBMC and 293T-Jurkat cell datasets are available from https://support.10xgenomics.com/single-cell-gene-expression/datasets. The intestine dataset can be assessed at the GEO under accession code GSE123516. Moreover, the accession codes of other datasets are: the human hippocampus scRNA-seq dataset (GSE131258), the Drosophila wing disc datasets (GSE155543), the mouse embryonic stem cells dataset (GSE65525), the mouse somatosensory cortex dataset (GSE60361), the Jurkat dataset (GitHub: princethewinner/FiRE/data), the Bacillus subtilis cells dataset (GSE151940) and the mouse tracheal epithelial cells dataset (GSE103354).

Field-specific reporting

Please select the one below that is the best fit for your research. If you are not sure, read the appropriate sections before making your selection.

☒ Life sciences ☐ Behavioural & social sciences ☐ Ecological, evolutionary & environmental sciences

For a reference copy of the document with all sections, see nature.com/documents/nr-reporting-summary-flat.pdf

Life sciences study design

All studies must disclose on these points even when the disclosure is negative.

Sample size	No sample-size calculation was performed. First of all, all public Single cell RNA-seq datasets used in this study comprise thousands of cells. Furthermore, hypothesis testing is not necessary for our algorithm, thus the sample size is not a concern for data preprocessing and analysis
Data exclusions	In the experiment for performance evaluation and the second application, the processed 68 k PBMC dataset was represented as UMI counts, where genes that were expressed in less than three cells were excluded, leaving 20,387 genes for further analysis, and cells expressing less than 200 genes were also excluded, the exclusion criteria were pre-established by the contributor of the dataset. In the first application experiment, the intestine dataset was represented as UMI counts. Genes that were expressed in less than three cells were excluded, leaving 16,091 genes for further analysis. Cells expressing less than 200 genes were excluded, the exclusion criteria were pre-established by the contributor of the dataset. Of note, the exclusion criteria for eliminating low quality cells can be different based on the sequencing depth and platform of scRNA-seq datasets, we thus have used the exclusion criteria provided by the contributor of each dataset in the original research.
Replication	First of all, to mimic the rare cell phenomenon systematically, we diluted between 2 and 100 rare cells in major cell types with a wide range of rare cell proportions using simulation dataset by Splatter and the 68 k PBMC dataset to demonstrate the stability of our method. GapClust provides higher and more robust accuracy in rare cell detection than the other four methods. Especially in experimental datasets with only two rare cells, we generated 20 random datasets for replication in each rare cell proportion. And GapClust still outperform the other four methods. For sensitivity analysis, 100 replicates for each iteration were performed to obtain robust estimation for all competing approaches in three settings with different numbers of rare cell. Again, GapClust can offer higher accuracy with a few differentially expressed gene between the rare cell type and the abundant cell type. Taken together, GapClust provide better performance successfully at replication.
Randomization	Randomization is not relevant to our study. As there is no hypothesis testing between cell groups when searching for rare cells in our algorithm. Moreover, each single cell RNA-seq dataset was collected from same patient, and the there is no covariate information.
Blinding	Blinding is not relevant to our study. As there is no hypothesis testing between cell groups when searching for rare cells in our algorithm.

Reporting for specific materials, systems and methods

We require information from authors about some types of materials, experimental systems and methods used in many studies. Here, indicate whether each material, system or method listed is relevant to your study. If you are not sure if a list item applies to your research, read the appropriate section before selecting a response.

Materials & experimental systems

n/a	Involved in the study
☒	☐ Antibodies
☒	☐ Eukaryotic cell lines
☒	☐ Palaeontology and archaeology
☒	☐ Animals and other organisms
☒	☐ Human research participants
☒	☐ Clinical data
☒	☐ Dual use research of concern

Methods

n/a	Involved in the study
☒	☐ ChIP-seq
☒	☐ Flow cytometry
☒	☐ MRI-based neuroimaging
